# A New Strategy to Inhibit Scar Formation by Accelerating Normal Healing Using Silicate Bioactive Materials

**DOI:** 10.1002/advs.202407718

**Published:** 2024-09-28

**Authors:** Zhaowenbin Zhang, Chen Fan, Qing Xu, Feng Guo, Wenbo Li, Zhen Zeng, Yuze Xu, Jing Yu, Hongping Ge, Chen Yang, Jiang Chang

**Affiliations:** ^1^ Joint Centre of Translational Medicine The First Affiliated Hospital of Wenzhou Medical University Wenzhou 325000 P. R. China; ^2^ Zhejiang Engineering Research Center for Tissue Repair Materials Wenzhou Institute University of Chinese Academy of Sciences Wenzhou 325000 P. R. China; ^3^ State Key Laboratory of High‐Performance Ceramics and Superfine Microstructure Shanghai Institute of Ceramics Chinese Academy of Sciences Shanghai 200050 P. R. China; ^4^ Department of Plastic Surgery Shanghai Jiaotong University Affiliated Sixth People's Hospital Shanghai 200050 P. R. China

**Keywords:** bioactive material, bioglass, human skin dermal fibroblast, hypertrophic scar fibroblasts, scar

## Abstract

Inspired by the scar‐free wound healing in infants, an anti‐scar strategy is proposed by accelerating wound healing using silicate bioactive materials. Bioglass/alginate composite hydrogels are applied, which significantly inhibit scar formation in rabbit ear scar models. The underlining mechanisms include stimulation of Integrin Subunit Alpha 2 expression in dermal fibroblasts to accelerate wound healing, and induction of apoptosis of hypertrophic scar fibroblasts by directly stimulating the N‐Acylsphingosine Amidohydrolase 2 expression in hypertrophic scar fibroblasts, and indirectly upregulating the secretion of Cathepsin K in dermal fibroblasts. Considering specific functions of the bioactive silicate materials, two scar treatment regimes are tested. For severe scars, a regenerative intervention is applied by surgical removal of the scar followed by the treatment with bioactive hydrogels to reduce the formation of scars by activating dermal fibroblasts. For mild scars, the bioactive dressing is applied on the formed scar and reduces scar by inducing scar fibroblasts apoptosis.

## Graphical abstract

Drawing inspiration from the remarkable scar‐free healing of skin wounds in infants, a 45S5 bioglass/sodium alginate composite hydrogel (BG/SA) is designed to mimic the wound‐healing microenvironment of infant skin. This composite hydrogel enhances normal dermal fibroblast viability and migration and promotes hypertrophic scar fibroblast apoptosis via SiO_3_^2‐^, effectively reducing the formation of scars.

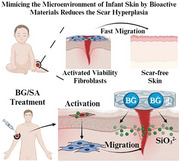

## 1 Introduction

When skin sustains severe injuries, such as burns or chemical corrosion, the dermis layer can be destroyed, leading to the formation of hard and irregular hypertrophic scars.^[^ ^1^ ^]^ These scars not only detract from the aesthetic appearance of the skin but also result in the loss of its natural functionality, including the disappearance of skin appendages like hair follicles and sweat glands.^[^ ^2^ ^]^ Additionally, patients may endure various complications, including itching and pain.^[^ ^3^ ^]^ In clinical practice, due to the difficulty in completely removing hypertrophic scar fibroblasts (HSF, referring to myofibroblasts in scar tissue) through a single surgical procedure, staged excision is often employed.^[^ ^4^ ^]^ This approach involves multiple surgeries spaced several weeks or months apart to remove the scar, or it may necessitate the use of anti‐scar medications as an adjunct therapy.^[^ ^5^ ^]^ However, this staged excision method significantly increases the patient's treatment burden. Commonly used anti‐scar medications, such as 5‐fluorouracil (5‐FU), bleomycin, tranilast, and botulinum toxin, primarily function by inducing HSF apoptosis. However, they have side effects such as damaging normal skin cells including epithelial cells, endothelial cells, and hair follicle cells, which may trigger more severe scar formation.^[^ ^1c^ ^]^

Interestingly, infants exhibit remarkable scar‐free healing during the wound repair process.^[^ ^6^ ^]^ The reasons for this phenomenon include higher activity of dermal fibroblasts, fewer HSF, milder inflammatory response, and higher collagen orientation compared to adults.^[^ ^6^ ^]^ Among these factors, dermal fibroblasts play a pivotal role in scar formation, because dermal fibroblasts with low vitality exhibit inadequate proliferation and migration capabilities during wound healing, preventing them from reaching the wound site promptly.^[^ ^7^ ^]^ Consequently, these cells differentiate into HSF as an alternative healing mechanism.^[^ ^8^ ^]^ However, these HSF overproliferate and stimulate local inflammatory responses, which ultimately leads to scar formation.^[^ ^4c^ ^]^ In contrast, dermal fibroblasts in infant skin exhibit high activity and robust proliferation and migration abilities,^[^ ^9^ ^]^ enabling them to rapidly reach the wound site and facilitate wound healing without scar formation. This also explains why the HSF content in infant wounds is significantly lower than that in adults, and the inflammatory response is less pronounced.^[^ ^4^, ^9^ ^]^ Therefore, inspired by the wound healing mechanism of infants, we believe that activating the proliferation and migration activity of dermal fibroblasts and mimicking the microenvironment of infant skin may help inhibit the formation of hypertrophic scars.

**Figure 1:**
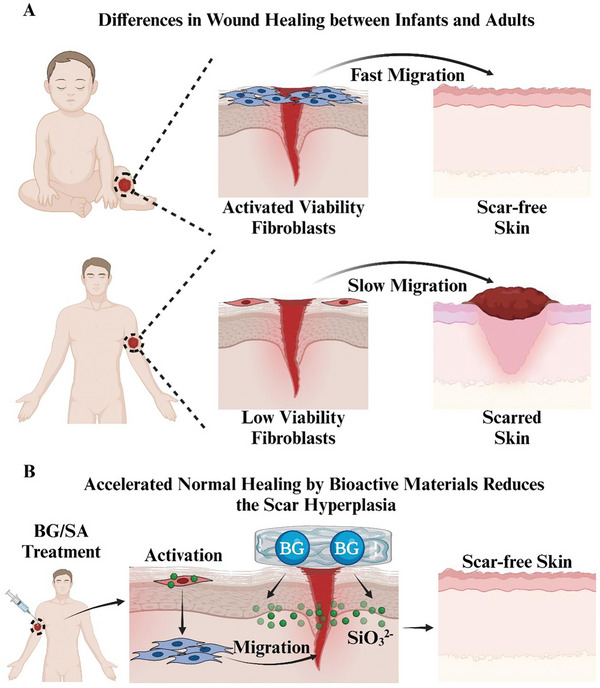
A) Differences in wound healing between infants and adults. In infants, when skin injury, fibroblasts activated spontaneously and rapidly migrated to the wound site, thereby accelerating wound healing, and inhibiting scar formation. Conversely, in adults, fibroblasts exhibit limited proliferation and migration activity, impeding wound healing and leading to scar formation. B) Accelerated normal wound healing by bioactive materials reduces the scar hyperplasia. Inspired by the scar‐free skin wound healing process in infants, a 45S5 bioglass/sodium alginate composite hydrogel (BG/SA) was used to accelerate normal wound healing through enhancing fibroblast migration by SiO_3_^2−^, thereby reducing scar formation.

However, most clinically used wound dressings fail to effectively prevent hypertrophic scars, as they promote wound healing primarily by activating myofibroblasts, which may aid in the healing of chronic wounds, but mature myofibroblasts may also result in the formation of scars.^[^ ^9^ ^]^ Previous studies have demonstrated that silicate bioactive materials can effectively promote wound healing, primarily by activating the proliferation and migration of dermal fibroblasts,^[^ ^10^ ^]^ and the collagen in the healed wounds exhibits a more optimized orientation.^[^ ^11^ ^]^ Furthermore, these materials could inhibit immune responses, thereby slowing down the fibrosis process in certain organs and tissues, although the underlying mechanisms remain unclear.^[^ ^12^ ^]^ Based on these preliminary findings, we hypothesize that silicate bioactive materials may create a regenerative microenvironment similar to infant skin wound, which is characterized by highly active dermal fibroblasts, low levels of HSF, and reduced inflammatory responses, and ultimately inhibit scar formation (**Figure** **1**).

To verify above hypothesis, we selected 45S5 Bioglass (BG), which is a well‐known silicate bioactive materials for tissue regeneration. The BG was incorporated in sodium alginate (SA) to form injectable composite hydrogels (BG/SA) with the consideration that the hydrogel system can facilitate bioactive ion release and create good wound‐healing environment.^[^ ^13^ ^]^ The effect of BG/SA in inhibiting scar formation in vivo was evaluated using rabbit ear hypertrophic scar models, and the possible biological mechanisms were explored by investigation of gene expression of BG‐stimulated dermal fibroblasts and HSF with the focus of the interactions between both cells in the presence of BG. Additionally, for potential applications of BG/SA, two treatment regimens such as a regenerative intervention and a direct treatment regime were evaluated for severe and mild hypertrophic scars, respectively.

## 2 Results

### 2.1 BG/SA Prevents Scars in Rabbit Ears Caused by Acid Corrosion

**Figure 2:**
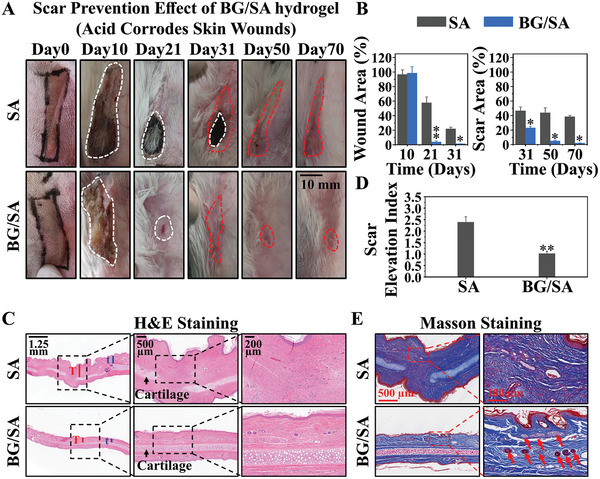
Prevention effect of BG/SA in a rabbit ear scar model in vivo. A) Photographs of wound/scar area after different treatments. (The white circle represents the area of the wound; the red circle represents the area of the scar.) B) Wound area and scar area percentage after different treatments (Statistics based on optical photos in Figure 2A.). C) H&E staining of skin sections on day 70 with different treatments (The red line: T: The thickness of scar dermis. The blue line: t: The thickness of normal skin dermis. The black arrow: cartilage.) D) Scar elevation index (Ratio of T/t) of skin sections on day 70 with different treatments (Statistics based on H&E staining in Figure 2C.). (E) Masson staining of skin sections on day 70 with different treatments (The red arrow: New born hair follicles). (*p < 0.05, **p < 0.01 compared with SA). (SA: The wounds were treated with SA from day 2 to day 70; BG/SA: The wounds were treated with BG/SA from day 2 to day 70.) Four experimental animals (two experimental animals per group) were used with two wounds created on each ear. *n* = 4 per group.

For the study of anti‐scar effect of bioactive materials, we used the typical bioactive material 45S5 bioglass to prepare a composite hydrogel (Figure S1, Supporting Information) and applied on an acid‐etched rabbit ear scar model. The wounds were treated with BG/SA on the second day after acid corrosion. It can be clearly observed from the optical photos that BG/SA significantly promoted the healing of skin wounds on the 21st day (marked with a white circle), whereas the wounds in the SA group had not yet healed by the 31st day (**Figure** **2**A).

It is interesting to see that, with faster healing, the area of scars formed in BG/SA group on the 31st day (marked with a red circle) was significantly less than that in the SA group (Figure 2A). Further quantitative analysis also proved that BG/SA more significantly promoted wound healing before 31 days and produced less scar tissue after 31 days as compared to SA (Figure 2B).

Rabbit ear tissues were collected for H&E and Masson section staining on day 21 and 70. On day 21, it was clearly observed that the epithelial layer of the skin had been completely repaired after treating with BG/SA. In contrast, the epithelial layer of the skin treated with SA alone remained unhealed (Figure S2, Supporting Information). On day 70, it was found from H&E section staining that the epidermal layer of the rabbit ears in the SA group was uneven, and the dermal layer thickness was inconsistent, representing uneven hypertrophic scars. In contrast, the epidermal layer of the rabbit ears in the BG/SA group was very smooth, and the entire dermal layer thickness was uniform (Figure 2C). Quantitative statistics of the scar index (scar dermal layer thickness T/normal dermal layer thickness t) is only 1.01, while the scar index of the SA group reached 2.39 (Figure 2D). In addition, Masson staining results showed that BG/SA also significantly promoted the regeneration of skin appendages (hair follicles) in the scar area, while almost no skin appendages were observed in the SA group (Figure 2E). Quantitative analysis further demonstrated that the number of new hair follicles in the BG/SA group was significantly higher than that in the SA group, indicating that BG/SA activates the normal skin repair process rather than the fibrotic repair process (Figure S3, Supporting Information).

### 2.2 Regulatory effect of BG on Human Skin Dermal Fibroblasts (HDF)

To explore the possible mechanisms for inhibitory effect of BG on scars, we first prepared BG extract and studied its impact on HDF viability and migration. The results showed that BG extract not only significantly enhanced HDF cell viability (Figure S4, Supporting Information) but also significantly promoted HDF migration (Figure S5, Supporting Information). Moreover, the optimal effective dilution ratio of the BG extract is 1/16, with a SiO_3_^2−^ ion concentration of 51.13 ± 0.92 ppm (Table S1, Supporting Information).

**Figure 3:**
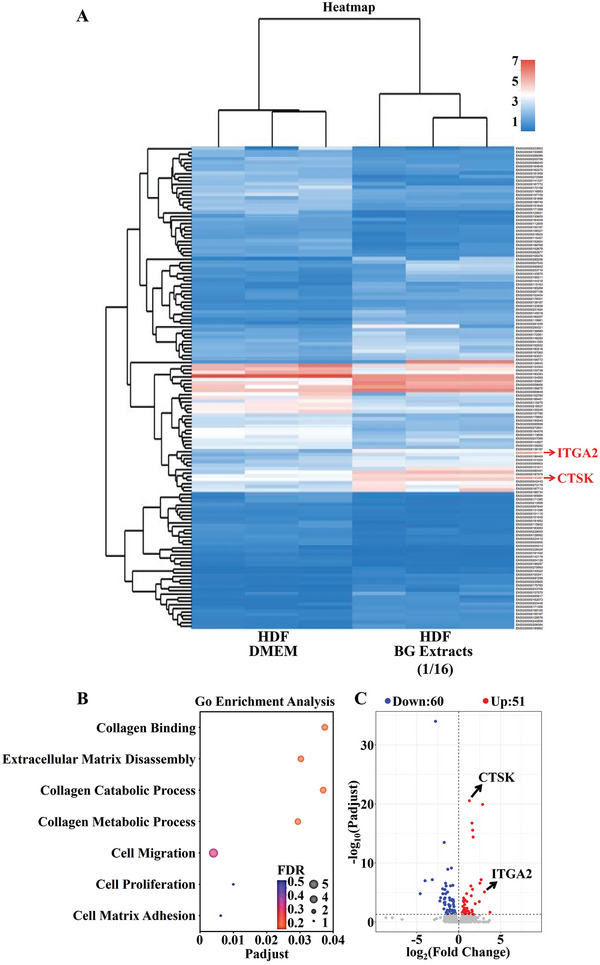
Sequencing analysis of HDF gene changes with or without BG extracts (1/16 dilution) treatment. A) The cluster heatmap of differential genes of HDF and BG‐treated HDF. B) Up‐Gene Ontology (GO) term types enriched by up‐regulated genes of HDF and BG‐treated HDF. C) Volcano plot of differential genes of HDF and BG‐treated HDF.

Further transcriptome sequencing analysis of BG stimulated HDF revealed that the gene expression of HDF induced by BG stimulation was significantly changed (**Figure** **3**A). Interestingly, the upregulated genes were mainly enriched in GO terms related to HDF activity including cell migration, cell proliferation, collagen binding, and extracellular matrix disassembly (Figure 3B). Among the enriched genes in these GO terms, Cathepsin K (CTSK) and Integrin Subunit Alpha 2 (ITGA2), which are associated with cell proliferation and migration, showed significant upregulation (Figure 3C). To further validate the expression of these two differentially expressed genes in HDF, we analyzed BG stimulated HDF using qPCR. The results confirmed that BG significantly activated CTSK and ITGA2 expression in HDF (Figure S6, Supporting Information). These findings suggest that BG may activate HDF, promote their proliferation and migration by upregulating CTSK and ITGA2, thereby rapidly promoting skin wound healing. To further verify the regulatory mechanism of BG on HDF, we designed siRNAs specifically targeting ITGA2 and CTSK to knock down the expression of these genes in HDF respectively and found that ITGA2 siRNA and CTSK siRNA did effectively inhibit the expression of ITGA2 and CTSK in HDF, respectively (Figure S7, Supporting Information). Additionally, CTSK siRNA and ITGA2 siRNA alone did not affect the viability, proliferation and migration ability of HDF (Figures S7–S9, Supporting Information). Then, when HDF was transfected with siRNAs of ITGA2, the activation effect of BG on HDF in cell viability and migration was clearly suppressed suggesting the critical role of this gene in BG activation. However, CTSK siRNA only slightly inhibited the enhancing effect of BG on HDF cell viability and migration suggesting that CTSK may not be the main factor for BG activation of HDF viability and migration (Figures S8 and S9, Supporting Information).

### 2.3 Comparison of Fetal Fibroblasts with Adult Fibroblasts

To further investigate whether BG has the potential to create a regenerative microenvironment similar to infant skin at the wound site, we conducted a comparison between fibroblasts derived from fetal mice and those derived from adult mice under BG activation. The results indicate that fetal fibroblasts exhibit significantly higher activity than adult fibroblasts, and BG activation notably enhances the activity of adult fibroblasts, achieving high cell viability and cell migration ability approaching the level of fetal fibroblasts (Figure S10, Supporting Information). Additionally, we observed higher α‐SMA expression in adult fibroblasts as compared to fetal fibroblasts, suggesting a higher transformation potential toward HSF. However, BG activation of adult mouse fibroblasts resulted in significant decrease of α‐SMA, which is comparable to those of fetal mouse fibroblasts, indicating that BG activation diminishes the ability of adult mouse fibroblasts to transform into HSF (Figure S11, Supporting Information). Furthermore, we compared the expression of inflammatory factors in fetal and adult fibroblasts and found that the expression of IL‐1β and TNF‐α in adult cells is significantly higher than that in fetal cells, and BG activation of adult cells significantly reduces IL‐1β and TNF‐α expression (Figure S11, Supporting Information). Notably, we discovered that the expression of ITGA2 and CTSK in adult fibroblasts is significantly lower than that in fetal fibroblasts, while BG activation notably increases the expression of ITGA2 and CTSK in adult fibroblasts (Figure S12, Supporting Information). These findings suggest that BG activation induces alterations in several key characteristics of adult fibroblasts, aligning them more closely with that of the fetal fibroblasts, such as cell viability, cell migration activity, and expression of inflammatory factors, which may contribute to creating a wound healing microenvironment similar to that of fetal wound healing.

### 2.4 Regulatory Effect of BG on Hypertrophic Scar Fibroblasts (HSF)

**Figure 4:**
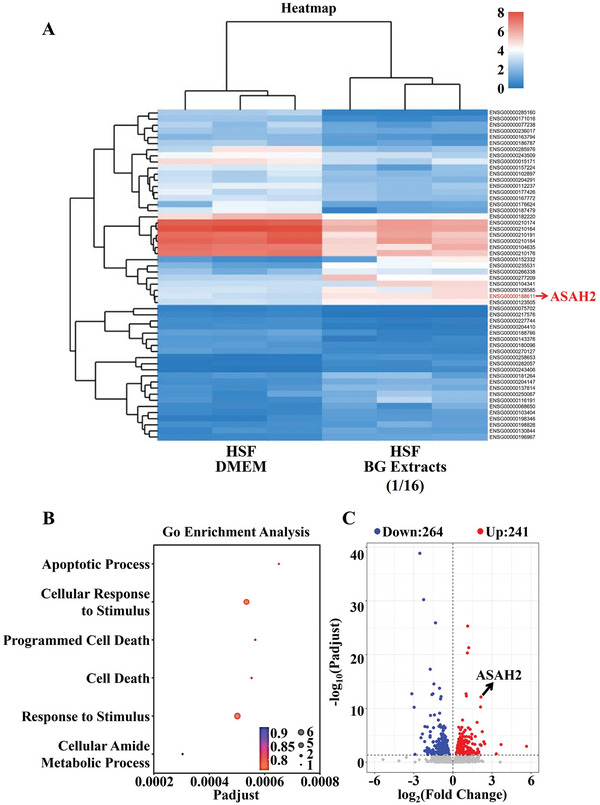
Sequencing analysis of HSF gene changes with or without BG extracts (1/16 dilution) treatment. A) The cluster heatmap of differential genes of HSF and BG‐treated HSF. B) Up‐Gene Ontology (GO) term types enriched by up‐regulated genes of HSF and BG‐treated HSF. C) Volcano plot of differential genes of HSF and BG‐treated HSF.

It is known that HSF plays a key role in scar formation.^[^ ^4^ ^]^ Therefore, we also investigated the effect of BG on HSF. First, we evaluated the expression of α‐SMA (a typical marker of HSF) to confirm the stability of the HSF phenotype (Figure S13, Supporting Information). Subsequently, we began to assess the regulatory effect of BG on HSF. We found that in comparison to the Blank group, BG led to a substantial decrease in the viability of HSF. Specifically, cell viability was reduced by ≈40.35% (stimulated by 1/16 BG) compared to the Blank group on the 5th day of culture (Figure S14, Supporting Information). In addition, BG treatment clearly reduced the migratory capacity of HSF, with a decline of ≈26.23% in comparison to the Blank group (Figure S5, Supporting Information). Due to the direct inhibitory effect of BG on HSF, we further conducted transcriptome sequencing analysis of BG‐treated HSF. **Figure** **4**A illustrates the effect of BG on HSF gene expression. Interestingly, these differentially expressed genes are mainly enriched in genes related to cell apoptosis, such as apoptotic process, cellular response to stimulus, and programmed cell death (Figure 4B). ASAH2 is one of the most significantly differentially expressed genes in GO terms enrichment analysis, and it is in the upper right corner of the volcano plot, indicating its significant upregulation (Figure 4C). To confirm the expression of ASAH2 in HSF, we analyzed BG‐stimulated HSF using qPCR. The results showed that BG indeed significantly upregulated ASAH2 expression in HSF (Figure S15, Supporting Information). Based on the above results, it is hypothesized that the direct inhibitory effect of BG on HSF is mediated through the regulation of ASAH2 expression in HSF cells. First, we designed a specific siRNA targeting ASAH2 to knock down its expression in HSF, and found that siRNA ASAH2 did effectively inhibit the expression of ASAH2 in HSF (Figure S16, Supporting Information). Additionally, ASAH2 knockdown had no significant effect on the number and viability of HSF (Figure S16, Supporting Information). Then, results showed that ASAH2 siRNA indeed significantly inhibited the ability of BG to induce HSF apoptosis (Figure S17, Supporting Information).

**Figure 5:**
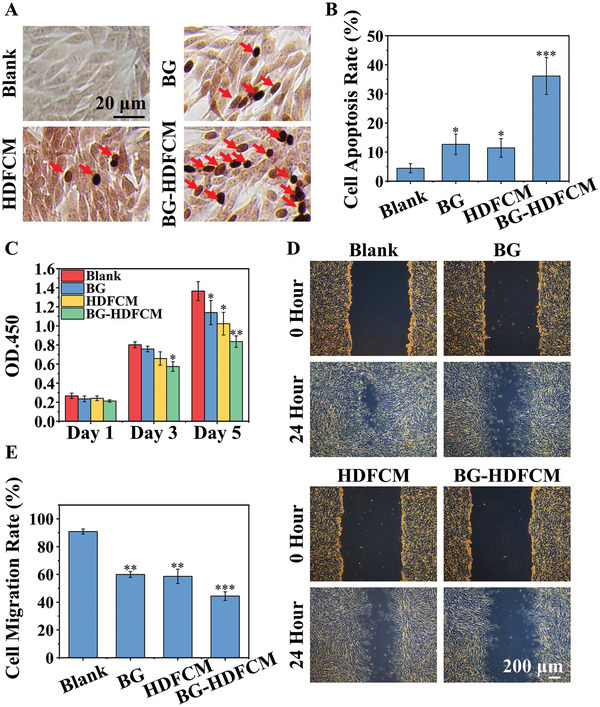
The inhibition of HSF by BG and HDF activated by BG. A) The effect of conditioned medium acquired from HDF cultured with a 1/16 dilution of BG (BG‐HDFCM) on HSF cell apoptosis (TUNEL staining). B) Quantitative analysis of apoptosis rate of HSF in TUNEL staining. C) The influence of BG‐HDFCM on HSF cell viability by CCK8 assay. D) Representative cell migration images of HSF cultured with BG‐HDFCM. E) The effect of BG‐HDFCM on HSF cell migration rate. (*p < 0.05, **p < 0.01, ***p < 0.001 compared with Blank). Blank: normal DMEM medium; BG: DMEM medium with a 1/16 dilution of BG; HDFCM: HDF normal conditioned medium; BG‐HDFCM: conditioned medium from HDF cultured with a 1/16 dilution of BG.

In addition to direct regulatory effect of BG on HSF, it is reasonable to consider the interaction of HDF and HSF in scar formation. Interestingly, previous study has revealed that the content of HSF in infant skin wounds is significantly lower than that in adults^[^ ^9^ ^]^ suggesting the possible role of the HDF activity on HSF inhibition. However, the effect of the activity of HDF on HSF is unknown. Therefore, we first used conditioned medium from HDF with or without BG activation to culture HSF and analyzed HSF apoptosis by TUNEL staining. Interestingly, we found that the conditioned medium from HDF without BG activation (HDFCM) induced apoptosis of HSF in certain degree, while the BG activated HDF (BG‐HDFCM) much more significantly induced the apoptosis of HSF as compared to HDFCM (**Figure** **5**A). Quantitative analysis further confirmed that under the stimulation of BG‐HDFCM, the number of apoptotic HSF was significantly higher than that in the HDFCM group and the BG group, while no significant difference was observed between the latter two groups (Figure 5B). In addition, BG‐HDFCM also resulted in a significant decrease in the viability and migration ability of HSF cells, with a reduction of cell viability (day 5) of ≈38.8% and migration ability (24 h) of ≈51.15% compared with Blank group (Figure 5C–E). Interestingly, we observed that the inhibitory effect of the conditioned medium derived from BG activated HDF (BG‐HDFCM) on HSF apoptosis was significantly suppressed by transfection of HDF with CTSK siRNA, indicating the critical role of CTSK gene in indirect induction of HSF apoptosis by BG activation of HDF (Figure S18, Supporting Information).

### 2.5 Therapy for Severe Scars by Wound Healing Acceleration using Bioactive BG/SA

**Figure 6:**
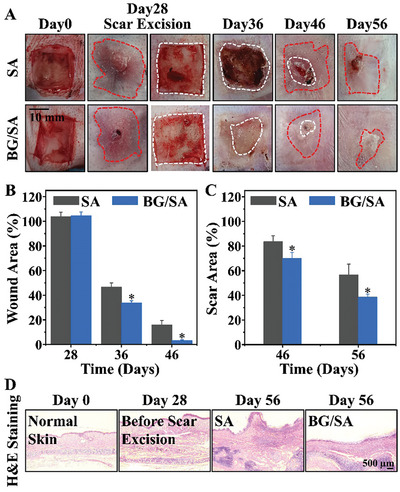
Treatment Effect of BG/SA in a severe rabbit ear scar model in vivo. A) Photographs of wound/scar area after different treatments. The white circle represents the area of the wound; the red circle represents the area of the scar. B) Wound area percentage after different treatments (Statistics based on optical photos in Figure 6A.). C) Scar area percentage after different treatments (Statistics based on optical photos in Figure 6A.). D) H&E staining of skin sections. (*p < 0.05 compared with SA) Four experimental animals (two experimental animals per group) were used with two wounds created on each ear. *n* = 4 per group.

Based on ability of BG to effectively activate HDF and rapidly promote wound healing, thereby reducing scar formation, we propose a regenerative intervention strategy for treating severe hypertrophic scars. This involves surgically removing the scars to create a fresh wound, and then treat with bioactive BG/SA during the wound healing process to avoid scar reformation by promoting rapid wound healing. We designed an experiment by first surgically removing the dermis of rabbit ears and exposing the cartilage layer to allow scar formation, and then we performed a second surgery to completely remove the scar tissue followed with BG/SA treatment. The results showed that, after the removal of the scar formed in first surgery, the application of BG/SA on the wound significantly reduced new scar formation. The new scar formed in BG treated wound (BG/SA treatment group) was significantly smaller as compared to the control group treated with SA alone and this difference became more pronounced over time (**Figure** **6**A). The wound healing rate in the BG/SA treatment group was also significantly faster on day 36 (8 days after BG/SA treatment). By day 46, the wounds in the BG/SA treatment group had mostly healed, leaving only a small amount of scabbing. Further quantitative analysis confirmed that BG/SA significantly accelerated wound healing from days 28 to 46 (Figure 6B) and significantly inhibited scar tissue formation from days 46 to 56 (Figure 6C). The quantitative results of H&E staining and Scar Index showed that the smoothness of the skin surface after BG/SA treatment was significantly restored, with a Scar Index value of 1, which is almost equivalent to the level of normal skin. In contrast, the Scar Index value before surgical removal was as high as 2.38. Additionally, the Scar Index value of the SA control group after the second surgical removal was 1.77, which was significantly higher than that of the BG/SA treatment group (Figure 6D; Figure S19, Supporting Information). Masson staining demonstrated that BG/SA stimulated the regeneration of hair follicles within scar tissues (Figure S20, Supporting Information). Quantitative analysis confirmed that the number of newly formed hair follicles in the BG/SA group was significantly higher than that in the SA group (Figure S21, Supporting Information). Furthermore, the results of scar tissue PCR analysis indicated that both ITGA2 and ASAH2 were significantly upregulated after BG/SA intervention. Notably, the upregulation of ITGA2 expression was more pronounced, which might be attributed to the activation of dermal fibroblasts by BG/SA after the second surgical excision (Figure S22, Supporting Information). Compared to SA, the significantly increased Vimentin‐positive staining in the BG/SA group indicates that BG/SA is indeed effective in promoting the activity of dermal fibroblasts in vivo (Figure S23, Supporting Information).

### 2.6 Therapy of Mild Scars by Inhibitory activity of BG on HSF

**Figure 7:**
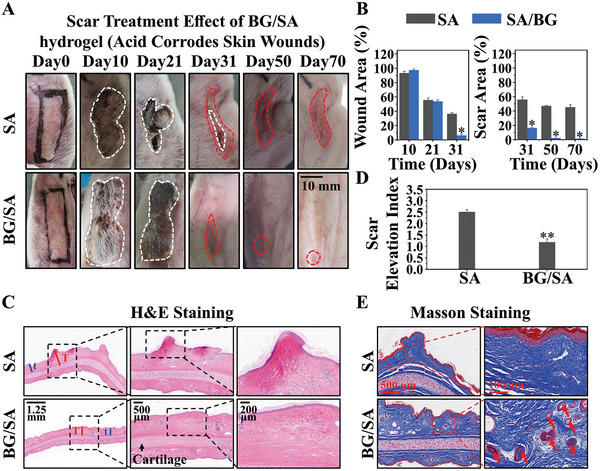
Treatment Effect of BG/SA in a mild rabbit ear scar model in vivo. A) Photographs of wound/scar area after different treatments. The white circle represents the area of the wound; the red circle represents the area of the scar. B) Wound area and scar area percentage after different treatments (Statistics based on optical photos in Figure 7A.). C) H&E staining of skin sections on day 70 with different treatments (The red line: T: The thickness of scar dermis. The blue line: t: The thickness of normal skin dermis. The black arrow: cartilage.) D) Scar elevation index (Ratio of T/t) of skin sections on day 70 with different treatments (Statistics based on H&E staining in Figure 7C.). (E) Masson staining of skin sections on day 70 with different treatments (The red arrow: New born hair follicles). (*p < 0.05, **p < 0.01 compared with SA). (SA: The wounds were treated with SA from day 22 to day 70; BG/SA: The wounds were treated with BG/SA from day 22 to day 70.) Four experimental animals (two experimental animals per group) were used with two wounds created on each ear. *n* = 4 per group.

Based on the direct inhibitory effect BG on HSF, we further investigated the therapeutic effect of BG/SA on pre‐existing scars. We first induced scar formation on rabbit ears through acid corrosion for 21 days (Figure S24, Supporting Information). Then, we treated scars with BG/SA on day 22. The results showed that BG/SA significantly reduced the scar tissue (red circled area) on the surface of rabbit ears by day 31, and the scar tissue continued to shrink with BG/SA intervention (**Figure** **7**A,B). It is worth noting that the currently anti‐scar product, medical silicone gel, has minimal inhibitory effect on formed scars. Specifically, on the 70th day, the scar area in both the control group (Only Injury) and the silicone gel group exceeded 40% (Figures S25 and S26, Supporting Information). However, the scar area for BG/SA on the 70th day was less than 2% (Figure 7A,B). H&E staining results on day 70 revealed that, as compared to the scar tissue on day 21 (Scar Elevation Index = 1.79) (Figure S24, Supporting Information), the skin surface after BG/SA treatment recovered its smoothness, with a Scar Index of only 1.18. In contrast, the SA group showed the presence of numerous uneven keloids, with a Scar Index reaching 2.41 (Figure 7C,D). Additionally, Masson staining demonstrated that BG/SA stimulated the regeneration of skin appendages, specifically hair follicles, within scar tissues (Figure 7E). Quantitative analysis further confirmed that the number of newly formed hair follicles in the BG/SA group was significantly higher than that in the SA group (Figure S27, Supporting Information), indicating the therapeutic effectiveness of BG/SA in treating newly formed scar tissue.

Moreover, the results of scar tissue PCR analysis showed that both ITGA2 and ASAH2 were significantly upregulated after BG/SA intervention. Specifically, the upregulation of ASAH2 expression was more significant, possibly due to the inhibition of scar fibroblasts in the scar area by BG/SA (Figure S28, Supporting Information). In addition, immunohistochemical staining revealed that BG/SA treatment resulted in significant decrease of α‐SMA expression as compared to SA treatment, which further confirmed the effect of inhibition of HSF and excellent anti‐fibrotic ability of BG/SA in vivo (Figure S29, Supporting Information).

## 3 Discussion

**Figure 8:**
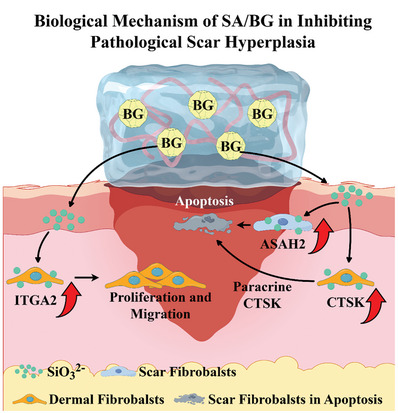
The mechanism of scar inhibition by BG/SA. BG/SA efficiently modulates the behavior of HDF and HSF through the release of SiO_3_^2−^ ions. By upregulating the expression of the ITGA2 gene, SiO_3_^2−^ enhances the proliferation and migration abilities of HDF, thereby increasing the healing rate of skin wounds. Simultaneously, HDF activated by SiO_3_^2−^ secrete cathepsin K (CTSK), leveraging this paracrine activity to induce the apoptosis of HSF. Additionally, SiO_3_^2−^ activates the apoptosis mechanism in HSF by upregulating the ASAH2 gene, further decreasing the HSF in scar and facilitating scar‐free skin healing.

The prevention and treatment of pathological scars in adults represent a significant clinical challenge, primarily because of delayed wound healing in adults after skin damage. Conversely, the dermal fibroblasts of infants exhibit high viability, facilitating rapid wound healing and typically resulting in scar‐free wound healing.^[^ ^6^ ^]^ Therefore, we propose a new strategy of using bioactive materials to activate dermal fibroblasts to accelerate wound healing and reduce scar formation or treat scars. We applied silicate‐based 45S5 bioglass/alginate composite hydrogel to test our hypothesis and found that the bioactive hydrogel indeed activate dermal fibroblasts and significantly reduce scar formation by promoting rapid wound healing (**Figure** **8**). According to our previous research findings, a key factor in the bioactivity of silicate materials lies in the release of SiO_3_^2−^ ions.^[^ ^1^, ^14^ ^]^ Specifically, in our previous wound repair studies, materials such as BG, calcium silicate, and forsterite were found to promote skin wound healing by releasing SiO_3_^2−^ ions.^[^ ^13^, ^15^ ^]^ Furthermore, research has also revealed that BG and calcium silicate can similarly inhibit fibrosis in other tissues, such as oral submucous fibrosis and pulmonary fibrosis, by releasing SiO_3_^2−^ ions.^[^ ^12^ ^]^ Based on these findings, we have reason to speculate that other silicate bioceramics may also have potential for treating skin scars. Therefore, comparison of different silicate bioactive materials in scar treatment is required for potential clinical application in future investigations. Previous studies have shown that BG can activate dermal fibroblasts, but it is unknown how this activation is regulated.^[^ ^10^, ^16^ ^]^ In this study, we found that BG significantly promotes the expression level of ITGA2 in HDF cells. Moreover, when the ITGA2 gene was knocked down by transfection of HDF with siRNA, the proliferative and migratory effects of BG on HDF cells were significantly inhibited, although the viability, proliferation and migration of the cells were not affected. These findings suggest that BG enhances the viability, proliferation and migration of dermal fibroblasts by upregulating the expression of ITGA2, thereby promoting scarless wound healing. These results suggest that ITGA2 might be a potential target for accelerating wound healing, in particular in the design of bioactive materials for scar‐free wound healing.

Theoretically, stimulation of dermal fibroblasts proliferation may also increase the risk of the conversion of dermal fibroblasts to HSF, thereby exacerbating scar formation and deterioration.^[^ ^17^ ^]^ However, it is unknown how dermal fibroblasts behaves in scar formation, and how dermal fibroblasts affects the fate of HSF. A previous study showed that the number of HSF in infant skin tissue during wound healing is significantly lower than that in adults.^[^ ^9^ ^]^ Considering the high activity of dermal fibroblasts in infants, it is likely that the activity of dermal fibroblasts may affect HSF apoptosis. Interestingly, we found in our study that BG indeed can induce HSF apoptosis. In particular, the inhibition is through two different ways. One is direct inhibitory effect when HSF cultured directly with BG extract solution. It is worth noting that this direct apoptotic effect is highly specific, targeting only HSF and has no significant impact on other skin‐related cells, such as dermal fibroblasts and hair follicle cells. We observed formation of mature hair follicle tissues in the dermal layer of skin treated with BG. Traditional anti‐scar medications primarily induce cell apoptosis by upregulating TNF‐α‐related signaling pathways, which are broad‐spectrum apoptotic signaling pathways.^[^ ^18^ ^]^ Almost all tissue cells enter the apoptotic pathway after high expression of TNF‐α.^[^ ^19^ ^]^ However, in this study, we found through sequencing and PCR analysis that the upregulation of apoptosis‐related genes in HSF treated with BG did not include TNF‐α. The most significantly upregulated differential gene is a ceramide called ASAH2. Notably, when HSF were transfected with ASAH2 siRNA and ASAH2 expression was knocked down, the original effect of BG in inducing HSF apoptosis was significantly attenuated. Based on these findings, it is reasonable to speculate that BG may promote the apoptosis of HSF by upregulating ASAH2. Interestingly, the ASAH2 gene plays a key function in cell cycle arrest and growth regulation. Specifically, it has the capability to initiate the caspase apoptosis pathway intracellularly, activate autophagosomes, and consequently induce apoptosis. Notably, this gene exhibits elevated sensitivity specifically within tumor cells, and its modulation does not affect the normal function of healthy cells.^[^ ^20^ ^]^ Considering the similarities between scars and tumors such as lack of spontaneous regression, high recurrence rate, and excessive collagen production, and the fact that some cancer therapies have been found effective in scar treatment,^[^ ^21^ ^]^ we have reason to believe that this ASAH2 gene might be a potential target for scar treatment, although this assumption needs to be confirmed by further investigation.

In addition to the direct apoptosis induction of HSF by BG, we discovered that the activation of dermal fibroblasts by BG also resulted in apoptosis of HSF. Our finding of the inhibitory function of highly active dermal fibroblasts on HSF apoptosis may explain the fact why the number of HSF is lower than that in adults.^[^ ^22^ ^]^ Furthermore, this result aligns with a recent study that challenges traditional beliefs. Traditionally, it is believed that healthy skin cells at the site of skin wound may mutate and form tumorigenic cells during proliferation, increasing the risk of skin cancer. However, a recent study revealed that promoting the proliferation of healthy skin cells can significantly inhibit tumorigenic cells.^[^ ^22^ ^]^ In our study, we observed similar phenomenon, in which BG activation of dermal fibroblasts inhibited HSF. More specifically, activated dermal fibroblasts induced the apoptosis of HSF. Interestingly, our results indeed showed that the gene expression level of CTSK was significantly increased in proliferative state of dermal fibroblasts, and its expression is significantly enhanced by the activation of BG. Intriguingly, CTSK is known to be primarily responsible for degrading excessive proteins in the body, such as excessive collagen responsible for scar formation,^[^ ^23^ ^]^ and massive degradation of collagen could directly lead to the apoptosis of HSF.^[^ ^24^ ^]^ Our results suggest that the scar‐inhibitory effect of BG may also be attributed to the regulation of CTSK gene expression by BG, since the CTSK siRNA transfection clearly suppressed the inhibitory effect of BG‐activated dermal fibroblasts on HSF apoptosis.

Considering our findings that BG/SA can significantly enhance the cell viability and migration of dermal fibroblasts and induce HSF apoptosis, we proposed two potential treatment regimes for scar therapy. For severe scars, which are difficult to reverse to normal skin, we proposed a regenerative intervention approach and consider to utilize the activity of BG/SA to activate dermal fibroblasts and accelerate wound healing. We hypothesize that if we completely remove the scar, and then apply bioactive wound dressing to inhibit new scar formation during the healing, the formation of new scars may be significantly reduced. Our results successfully demonstrated that bioactive BG/SA indeed significantly reduced scar re‐formation after surgical removal of the original scars. Furthermore, it is known that the recurrence rate of the scar is ≈80% after traditional scar removal therapies, including surgery, pressure therapy, laser treatment, and cryotherapy,^[^ ^25^ ^]^ and this high recurrence rate is closely related to the HSF content of the skin after wound healing.^[^ ^9^ ^]^ Therefore, our bioactive silicate‐based approach might also have the potential to reduce the scar recurrence rate, although this requires further investigation. In addition, for mild scars that has not fully matured, we consider to utilize the inhibitory function of BG/SA to HSF, which specifically induces HSF apoptosis in scars without damaging normal skin cells. Our results confirmed the effectiveness of the BG/SA in direct treatment of mild scars, which provides a new possibility for mild scar treatment. This strategy overcomes the indiscriminate damage to skin cells (including epithelial cells, endothelial cells, and hair follicle cells) caused by traditional anti‐scar drugs.^[^ ^25a–c^ ^]^ and avoids the risk of secondary skin damage.^[^ ^1a,c^ ^]^ Our results confirmed the effectiveness of the BG/SA in direct treatment of mild scars, which provides a new possibility for mild scar treatment.

## 4 Conclusion

Based on the concept of accelerating normal wound healing to reduce scar formation, this study revealed that the silicate‐based bioactive composite hydrogel exhibits excellent scar‐free wound healing effects in vivo. The scar inhibition activity of the composite hydrogel lies in the bioactive BG, which significantly activated dermal fibroblasts and promotes apoptosis of HSF cells. Gene knockdown results indicate that BG activation of dermal fibroblasts is primarily through the upregulation of ITGA2, whereas the promotion of HSF cell apoptosis is primarily through the upregulation of ASAH2. Additionally, BG indirectly induces apoptosis of HSF via paracrine behavior by upregulating the expression of CTSK in dermal fibroblasts. Therefore, this bioactive composite hydrogel can not only directly treat mild scars but also has the potential to treat severe scars through regenerative intervention after excision. In summary, bioactive silicate materials with the ability to activate fibroblasts and inhibit HSF provide a new strategy for scar‐free wound healing and scar treatment through their unique cell regulation mechanism.

## 5 Experimental Section

### The Preparation of 45S5 Bioglass (BG)

The SiO_2_ (Sinopharm Chemical Reagent, 20 035 717), CaO (Sinopharm Chemical Reagent, 10 005 928), P_2_O_5_ (Sinopharm Chemical Reagent, 10 015 528), and Na_2_O (Sinopharm Chemical Reagent, L03671225G) were uniformly mixed in a molar ratio of 45%:24.5%:6%:24.5% and placed in a platinum crucible. Subsequently, the mixed powder was gradually heated to a melting temperature of 1300 °C. Afterward, the high‐temperature melt was rapidly quenched. The quenched block was broken into small pieces and then ground using a ball mill. Finally, the desired 45S5 bioglass powder was obtained by sieving through a 400‐mesh screen.

### BG Extracts Preparation

The ion extracts of BG were prepared according to the previously reported protocol, which was adapted from the standard procedure in ISO10993‐1.^[^ ^26^ ^]^ Briefly, BG powders were added into serum‐free Dulbecco modified Eagle medium (DMEM) (Gibco, 11 965 092) at a solid/liquid ratio of 200 mg mL^−1^ and incubated at 37 °C for 24 h. The mixture was centrifuged at 8000 rpm for 10 min, and then the supernatant was sterilized through a filter membrane (Millipore, 0.22 µm) to obtain BG extracts. For further use, BG extracts were diluted with DMEM medium (with 10% fetal bovine serum (FBS, Gibco, 10 091 148) and 1% penicillin‐streptomycin (P/S, Gibco, 15 140 122)) at ratios of 1/2, 1/4, 1/8, 1/16, 1/32, 1/64, 1/128 and 1/256, respectively. The concentrations of Ca and Si ions in the extracts were detected by inductively coupled plasma atomic emission spectroscopy (ICP‐AES, Varian 715‐ES, USA).

### Cell Culture

Hypertrophic scar fibroblasts (HSF, model number: HUM‐iCell‐s035) and human skin dermal fibroblasts (HDF, model number: HUM‐iCell‐s001) were purchased from iCell (Shanghai) Biotechnology Co., Ltd. Both HSF and HDF were grown in 10% FBS (Gibco, 10 091 148)/DMEM (Gibco, 11 965 092) medium in a 5% CO_2_ atmosphere at 37 °C. Culture medium was changed on alternate days and cells were passaged when confluent. Cells from passages 4 through 8 were used in following experiments.

### Cell Viability Assay

Effects of BG extracts on the cell viability of HSF and HDF were evaluated using CCK‐8 assay (Cell counting kit‐8, Dojindo, Japan). Briefly, HSF and HDF were seeded in 96‐well plates at the density of 3 × 10^3^ cells per well and cultured with DMEM medium in a humidified 37 °C/5% CO_2_ incubator. After 24 h, the culture medium was replaced by the BG extracts at different dilution ratio (1‐1/256), and then further cultured for another 24, 48, or 72 h. A CCK‐8 assay was applied to evaluate cell viability according to the manufacture instruction. The absorbance of CCK‐8 liquid at wavelength of 450 nm was measured using a multimode microplate reader (Tecan Spark, Switzerland).

After 48 h incubation, HSF and HDF were rinsed with PBS and fixed with 4% paraformaldehyde. The cellular cytoskeleton was then stained with fluorescein isothiocyanate (FITC)‐phalloidin (Cytoskeleton Inc., USA) and the nuclei was stained with 4′,6‐diamidino‐2‐phenylindole (DAPI) (5 mg mL^−1^, Cytoskeleton Inc., USA), respectively. Confocal images were obtained by an inverted light microscope (Leica, DMI 3000B, German).

To further investigate the effects of BG extract on cells, a series of rigorous experiments were conducted. Initially, HDF with BG extract was cultured for 24 h and subsequently collected the conditioned medium to cultivate HSF. Additionally, to establish an effective control, conditioned medium was employed from HDF cultured in DMEM. To assess the viability of HSF, the CCK8 assay was utilized. To explore the migratory capabilities of HSF, a scratch assay was performed. Lastly, the apoptotic status of HSF was analyzed through TUNEL staining. This comprehensive experimental design aims to fully elucidate the specific impacts of BG extract on HSF cells.

### Comparison of Adult Mouse and Fetal Mouse Fibroblasts with/without BG Activation

Adult mouse fibroblasts (MIC‐iCell‐s010) were purchased from iCell (Shanghai) Biotechnology Co., Ltd., and fetal mouse fibroblasts (SNL‐025) were obtained from Wuhan Sean Biotechnology Co., Ltd. Briefly, both adult mouse fibroblasts and fetal mouse fibroblasts were seeded in 96‐well plates at a density of 3 × 10^3^ cells per well and cultured in DMEM medium in a humidified incubator at 37 °C with 5% CO_2_. After 24 h, the culture medium was replaced with 1/16 BG extracts, and then further cultured for another 72 h. A CCK‐8 assay was used to evaluate cell viability according to the manufacturer instructions. The absorbance of the CCK‐8 solution at a wavelength of 450 nm was measured using a multimode microplate reader (Tecan Spark, Switzerland). Furthermore, cells were cultured at a density of 1.5 × 10^5^ cells per well in a 6‐well plate using DMEM medium supplemented with 1/16 dilution of BG extracts. After 12 h of incubation, the cells were stained with crystal violet (Sigma, CAS:548‐62‐9) for 1 min, and optical images were captured using an optical microscope. The migration rate was analyzed using ImageJ software by calculating the ratio of the initial scratch area to the final scratch area. The α‐SMA, IL‐1β, TNF‐α, ITGA2, and CTSK expression was determined by PCR. The sequences of specific primers are summarized in Table S2 (Supporting Information).

### Sequencing

RNA sequencing was performed by Origingene Biomedical Technology Co., Ltd. (Shanghai, China). The RNA extraction and cNDA synthesis were performed as mentioned above, cDNA was then sequenced on an Illumina Hiseq X‐Ten (LC Bio, China). FastQC v.0.11.4 software was used to evaluate the raw data and obtain the clean reads. The reads were mapped to human genome GRCz11 using HISAT2 as previously published method.^[^ ^27^ ^]^ Gene expression was quantified by calculating the reads per kilobase transcriptome per million mapped reads (RPKM). Genes with fold change > 1 and false discovery rate (FDR) < 0.05 were considered as significant difference. Gene ontology (GO) analysis was performed using singular enrichment analysis.

### Assessment of the HSF Phenotype

HSF cells were cultured in a 6‐well plate at a density of 1.5 × 10^5^ cells per well using DMEM medium. HDF cells were used as a control group. After 3 days, total RNA was extracted using Trizol reagent (Invitrogen, Carlsbad, USA). Following determination of the RNA concentration and cDNA synthesis, the quantitative expression of targeted genes was measured using a Step One Plus Real‐Time PCR System (Applied Biosystems, USA). The sequences of specific primers for α‐SMA and β‐action are summarized in Table S2 (Supporting Information).

### Gene Expression

The gene expression in HDF and HSF after BG stimulation was determined as follow. Briefly, HDF and HSF were cultured at the density of 1.5 × 10^5^ cells per well in 6‐well plate with 1/16 dilution of BG extracts and DMEM medium. After 3‐day, total RNA was extracted using Trizol reagent (Invitrogen, Carlsbad, USA). After determination of the RNA concentration and cDNA synthesis, quantification expression of targeted genes was measured using a Step One Plus Real‐Time PCR System (Applied Biosystems, USA). The sequences of specific primers for ITGA2, CTSK, ASAH2, and β‐actin are summarized in Table S2 (Supporting Information).

### Transfection of Cells with siRNA

The knockdown efficacy of ITGA2 siRNA and CTSK siRNA was verified by PCR. HDF were seeded at a density of 1.5 × 10^5^ cells per well in a 6‐well plate. The cells were treated with ITGA2 siRNA or CTSK siRNA (Guangzhou RiboBio Co., Ltd., China) for 48 h. Total RNA was extracted using Trizol reagent (Invitrogen, Carlsbad, USA). Following the determination of the RNA concentration and cDNA synthesis, the expression levels of the targeted genes were measured using a Step One Plus Real‐Time PCR System (Applied Biosystems, USA). The sequences of specific primers for ITGA2, CTSK, and β‐actin are summarized in Table S2 (Supporting Information).

To assess the knockdown efficacy of ASAH2 siRNA, PCR analysis was conducted. HSF cells were seeded at a density of 1.5 × 10^5^ cells per well in a 6‐well plate and subsequently treated with ASAH2 siRNA (Guangzhou RiboBio Co., Ltd., China) for a duration of 48 h. Total RNA was extracted using Trizol reagent (Invitrogen, Carlsbad, USA). After determining the RNA concentration and synthesizing cDNA, the expression levels of the targeted genes were quantified using a Step One Plus Real‐Time PCR System (Applied Biosystems, USA). The sequences of the specific primers for ASAH2 and β‐actin are provided in Table S2 (Supporting Information).

### Evaluation of the Effect of siRNA Transfection on Cell Proliferation and Migration

The impact of ITGA2 siRNA and CTSK siRNA on cell proliferation was verified by cell counting. HDF were seeded at a density of 1.5 × 10^5^ cells per well in a 6‐well plate and treated with ITGA2 siRNA or CTSK siRNA for 48 h. The cells were then digested with trypsin, and the cell number was quantitatively counted using a cell counting plate. Finally, the impact of ITGA2 siRNA and CTSK siRNA on cell viability was verified using the CCK‐8 assay. HDF were seeded at a density of 1 × 10^3^ cells per well in a 96‐well plate and treated with ITGA2 siRNA or CTSK siRNA for 48 h. The CCK‐8 assay was performed to evaluate cell viability according to the manufacturer's instructions. The absorbance of the CCK‐8 solution at a wavelength of 450 nm was measured using a multimode microplate reader (Tecan Spark, Switzerland).

To investigate the impact of ASAH2 siRNA on cell proliferation, cell counting was performed. HSF cells were seeded at a density of 1.5 × 10^5^ cells per well in a 6‐well plate and treated with ASAH2 siRNA for 48 h. Following treatment, the cells were digested with trypsin, and the number of cells was quantitatively counted using a cell counting plate. Lastly, the effect of ASAH2 siRNA on cell viability was assessed using the CCK‐8 assay. HSF cells were seeded at a density of 1 × 10^3^ cells per well in a 96‐well plate and treated with ASAH2 siRNA for 48 h. The CCK‐8 assay was carried out according to the manufacturer's instructions to evaluate cell viability. The absorbance of the CCK‐8 solution at a wavelength of 450 nm was measured using a multimode microplate reader (Tecan Spark, Switzerland).

After RNA knocked down, the cell migration was analyzed. The cell monolayer in each well was gently scraped with a 200 µL plastic tip and washed with PBS to create a scratch (0 h). After 12 h of incubation, the cells were stained with crystal violet (Sigma, CAS:548‐62‐9) for 1 min, and optical images were captured using an optical microscope. The migration rate was analyzed using ImageJ software by calculating the ratio of the initial scratch area to the final scratch area. HSF were cultured at a density of 1.5 × 10^5^ cells per well in a 6‐well plate using DMEM medium supplemented with 1/16 dilution of BG extracts.

### Effect of siRNA Transfection on Cell Apoptosis

After RNA knocked down, the cells were subjected to TUNEL staining, utilizing a staining kit obtained from Sigma‒Aldrich, St. Louis, MO, USA, to assess the apoptotic status of HSF. Additionally, to investigate the impact of HDF paracrine factors on HSF apoptosis, HSFs were cultured in the conditioned medium collected from BG‐treated HDF and knocked down ITGA2 to further validate these effects.

### The Preparation of BG/SA

BG (10% w/v) particles were dispersed into a stirred 1.5% (w/v) alginate aqueous solution, followed by 5% (w/v) Glucono delta‐lactone (Glu, Aladdin, CAS: 90‐80‐2) was added and dispersed into the solution as well. Gelation subsequently occurred with the addition of Glu of which the hydrolysis released Ca ions from BG to cross‐link SA and finally give BG/SA.^[^ ^28^ ^]^ For the control experiments, pure alginate gels (SA) were prepared by the addition of 0.1 m CaCl_2_ solution into SA solution.

The morphology of BG/SA and SA was observed using a scanning electron microscope (SEM, Hitachi S‐4800, Japan). To confirm the existence of BG particles at hydrogel, the element distribution of each layer was characterized using a SEM accessary energy dispersive spectrometer (EDS) system.

The ion release behavior of BG/SA and SA was investigated by adding 1 g hydrogel in Tris‐HCl (1 mol L^−1^, 10 mL) and incubated for 1, 3, 5, 7, 10 days, respectively. All solution was then taken out and centrifuged at 1.2 × 10^4^ rpm for 10 min. The concentrations of Ca and Si ions of the supernatant solution were analyzed by ICP‐AES.

### Acid‐Corroded Rabbit Ear Hypertrophic Scar Model

The animal experimental protocols in this study were approved by the Committee of Experimental Animal Administration of Shanghai Sixth People's Hospital affiliated to Shanghai Jiao Tong University School of Medicine, and in accordance with international ethics guidelines and the National Institutes of Health Guide concerning the Care and Use of Laboratory Animals (DWLL2024‐0616). Briefly, 12 New Zealand white female rabbits weighting 2.5–3 kg were anesthetized and operated under sterile conditions. Two rectangular wounds of 4 × 1 cm, avoiding the central ear artery and marginal ear veins, were created by daubing concentrated hydrochloric acid (12 mol L^−1^) twice on the ventral surface of each ear. The wounds were randomly divided into three groups: Blank Control group (the wounds injury only); SA Prevention group (the wound was treated with SA twice a week from day 2 after operation); BG/SA Prevention group (the wound was treated with BG/SA twice a week from day 2 after operation); Positive Therapy Control group (the wounds were treated with Silicone Gel twice a week from day 22 to day 70); SA Therapy group (the wound was treated with SA twice a week from day 22 after operation); BG/SA Therapy group (the wound was treated with BG/SA twice a week from day 22 after operation and the scar already formed on the wound section). SA and SA/BG were applied to the wound site and secured with 3 m medical tape (T8030C‐1), allowing the hydrogel to remain in place on the wound for 4 days, thereby ensuring the release of SiO_3_^2−^ ions in the wound area. On the 4th day, the hydrogel was replaced. Before replacement, the wound was wiped with gauze moistened with physiological saline to remove any residual hydrogel. After recovery from anesthesia, the rabbits were returned to their cages. Photos were taken on day 0, 10, 21, 31, 50, and 70 after operation to observe any external changes to the wounds. Areas with significant color differences from normal skin and without skin appendages were defined as scar areas.^[^ ^29^ ^]^ The scar area percentage was determined by comparing the scar area with the wound area on day 0 in each group.

### Rabbit Ear Hypertrophic Scar Model with Secondary Surgical Excision

The animal experimental protocols in this study were approved by the Experimental Animal Ethics Committee of the Animal Research and Ethics Committee of Wenzhou Institute of University of Chinese Academy of Sciences (WIUCAS21030121), and in accordance with international ethics guidelines and the National Institutes of Health Guide concerning the Care and Use of Laboratory Animals. In this experiment, 4 female New Zealand white rabbits weighing between 2.5 and 3 kg were selected, anesthetized them, and conducted surgical procedures under sterile conditions. The specific steps involved excising the dermal layer of skin on the ventral surface of rabbit ears to create four 1 × 1 cm square wounds. Following a 28‐day natural healing period, these wounds developed scar tissue of corresponding size. Afterward, these scars were resected to form new 1 × 1 cm square wounds and randomly assigned them to two groups: Control group (the wound was treated with SA twice a week); Therapy group (the wound was treated with BG/SA twice a week). SA and SA/BG were applied to the wound site and secured using 3 m medical tape (T8030C‐1), enabling the hydrogel to remain in place on the wound for 4 days and thus ensuring the release of SiO_3_^2−^ ions within the wound area. On the 4th day, the hydrogel was replaced, and the wound was wiped with gauze moistened with physiological saline to remove any residual hydrogel. To document the healing progression, the wounds were photographed on days 0, 28, 36, 46, and 56, comparing the scar area in each group to the original wound size on day 0 to calculate the percentage of scar tissue. Areas exhibiting significant color differences from normal skin and lacking skin appendages were defined as scar areas.^[^ ^29^ ^]^

### Morphologic Analysis

Rabbits in each group were sacrificed on postoperative day. The samples in each group were excised and fixed with 4% paraformaldehyde for 48 h, embedded by paraffin, cross sectioned along the tissue, and stained using hematoxylin eosin (H&E) and Masson's trichrome for histological analysis. Masson staining was used to analyze the number of new hair follicles in the scar tissue after treatment. Scar elevation index (SEI) was calculated based on the ratio of scar tissue height (H) (the accurate thickness from the epithelium to cartilage of the scar tissue) to the normal tissue height (h) (the accurate thickness from the epithelium to cartilage of the normal tissue).^[^ ^30^ ^]^ The number of hair follicle were counted in five randomly selected fields of the Masson‐stained slides.

### Immunohistochemical Staining

Tissue sections from each group were fixed with 4% paraformaldehyde for 48 h, embedded in paraffin, and sectioned transversely. Severe rabbit ear scar tissues were subjected to Vimentin (Abcam) immunohistochemical staining, while mild rabbit ear scar tissues were subjected to α‐SMA (Abcam) immunohistochemical staining. The proliferation of dermal fibroblasts was evaluated by quantitative analysis of Vimentin‐positive cells, and the inhibition of HSF was assessed by quantitative analysis of α‐SMA‐positive cells.

### Expression of ITGA2 and ASAH2 in Scar Tissues In Vivo

Scar tissues were removed from the rabbit ear tissue. Areas with significant color differences from normal skin and without skin appendages were defined as scar areas.^[^ ^29^ ^]^ Total RNA was extracted from rabbit ear scar tissue (20 mg) using Trizol reagent (Invitrogen, Carlsbad, USA). After determining the RNA concentration and synthesizing cDNA, the quantification expression of targeted genes was measured using a Step One Plus Real‐Time PCR System (Applied Biosystems, USA). The sequences of specific primers for ITGA2, ASAH2, and GAPDH are summarized in Table S2 (Supporting Information).

### Statistical Analysis

The data were presented as means ± standard deviation (SD) and analyzed using a one‐way analysis of variance with a post hoc test. Significant difference was considered when *p* < 0.05 (*), 0.01 (**), and 0.001 (***).

## Conflict of Interest

The authors declare no conflict of interest.

## Author Contributions

Z.Z., C.F., Q.X., F.G. and W.L. contributed equally to this work. Z.Z., Q.X., F.G., W.L. and J.C. drew up the plan, carried out the experiments, analyzed the experimental data, and wrote the manuscript. Z.Z., C.F., Q.X., W.L., Z.Z., Y.X., J.Y., and F.G. performed the experiments. Z.Z., C.F., C.Y., and J.C. participated in data analysis and discussed the data. Z.Z., C.F., C.Y., and J.C. initiated, designed, and supervised the study and revised the manuscript.

## Supporting information



Supporting Information

## Data Availability

The data that support the findings of this study are available from the corresponding author upon reasonable request.
