# Supplementary material for: A New Strategy to Inhibit Scar Formation by Accelerating Normal Healing Using Silicate Bioactive Materials
Source: Adv Sci (Weinh). 2024 Sep 28;11(43):2407718. doi: 10.1002/advs.202407718 (PMC11578354; doi:10.1002/advs.202407718)
Supplement: Supplementary file 1 — Supporting Information [file ADVS-11-2407718-s001.docx]

**Supporting information**


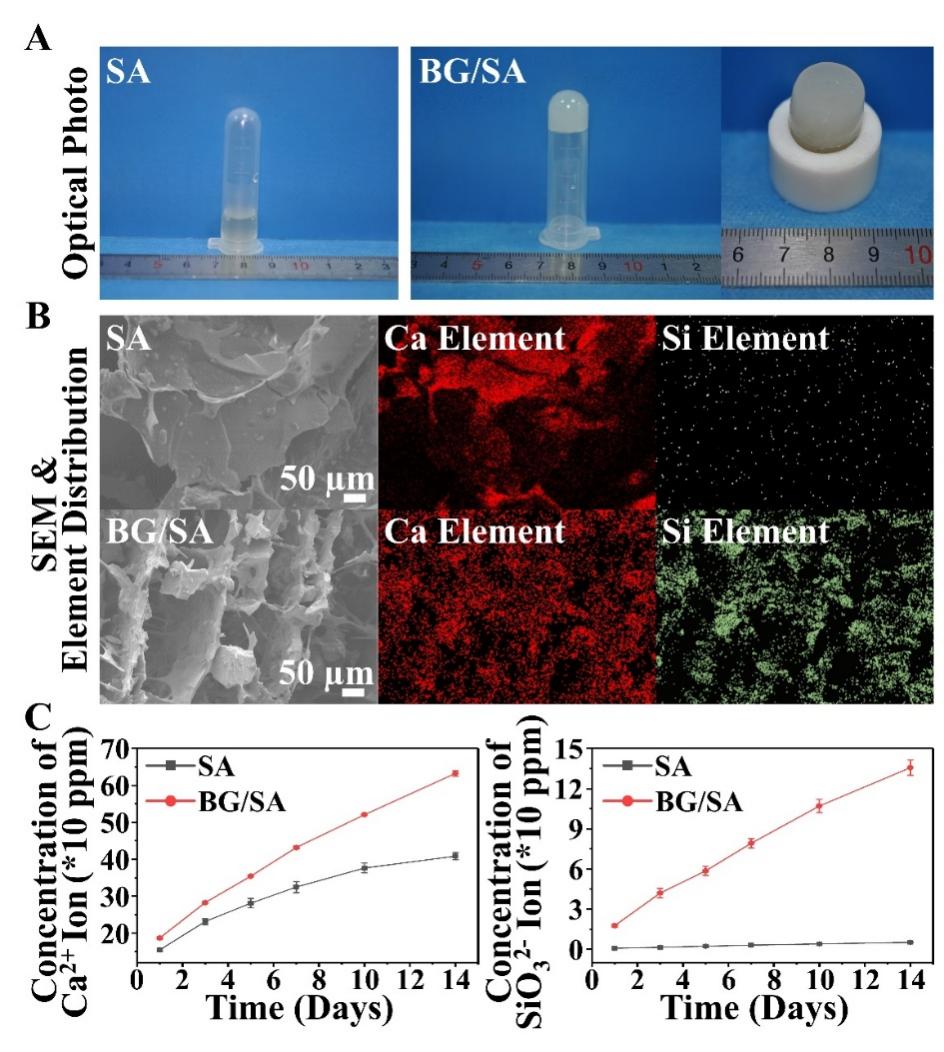


**Figure S1.** Characterization of composite hydrogels. (A) The optical photos of SA and BG/SA. (B) The SEM and element distribution (Ca and Si element) of SA and BG/SA. (C) The Ca^2+^ and SiO_3_^2-^ ions release from the BG/SA and SA.


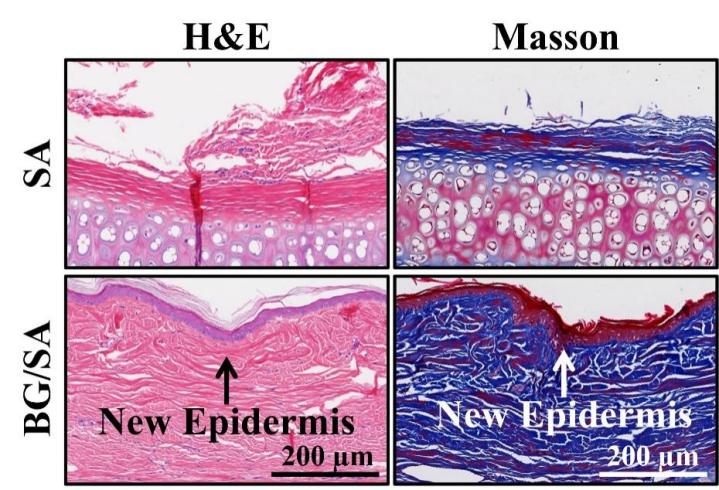


**Figure S2.** H&E and Masson staining of wounds sections on day 21 with BG/SA treatments.

(SA: The wounds were treated with SA from day 2 to day 70; BG/SA: The wounds were treated with BG/SA from day 2 to day 70.)


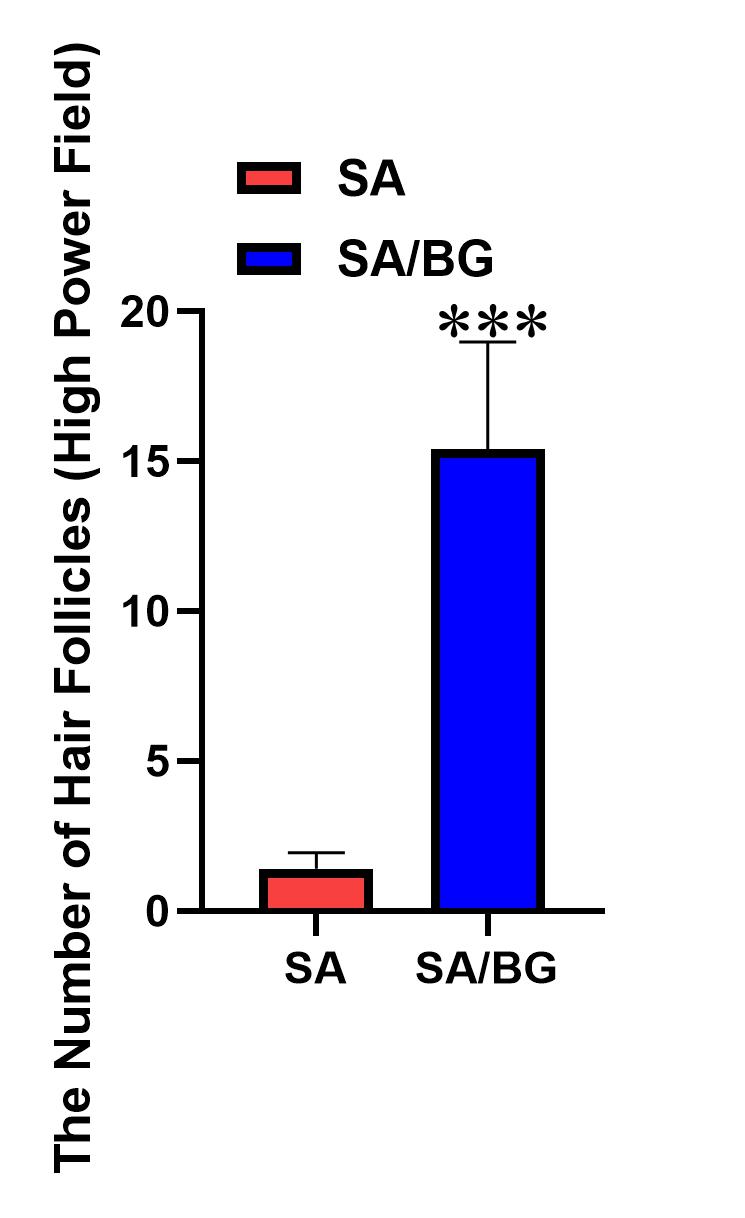


**Figure S3.** Quantitative analysis of new born hair follicles in the prevention of rabbit ear scar *in vivo* (***p < 0.001 compared with SA).


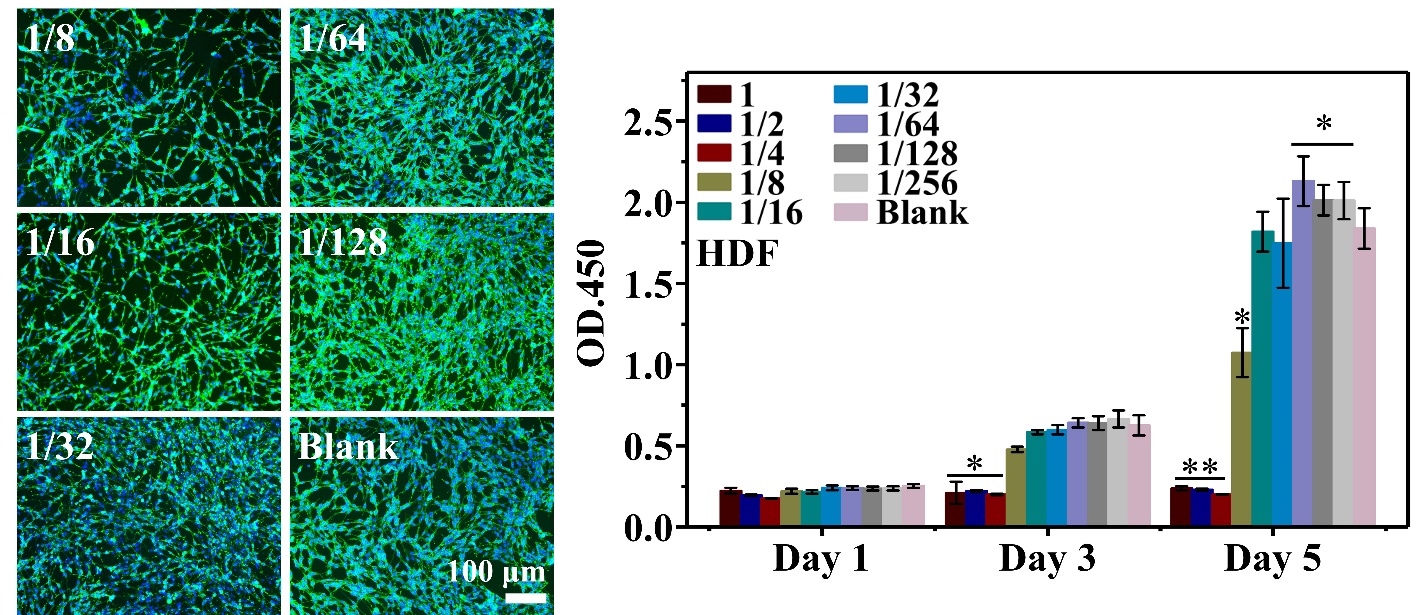


**Figure S4.** BG activates HDF viability. The cell viability of HDF cultured with BG extracts (1-1/256) at day 1, 3 and 5. Fluorescent images of HDF treated with BG extract (1/8-1/256) for 48 h. (*p <0.05, **p < 0.01 compared with Blank)


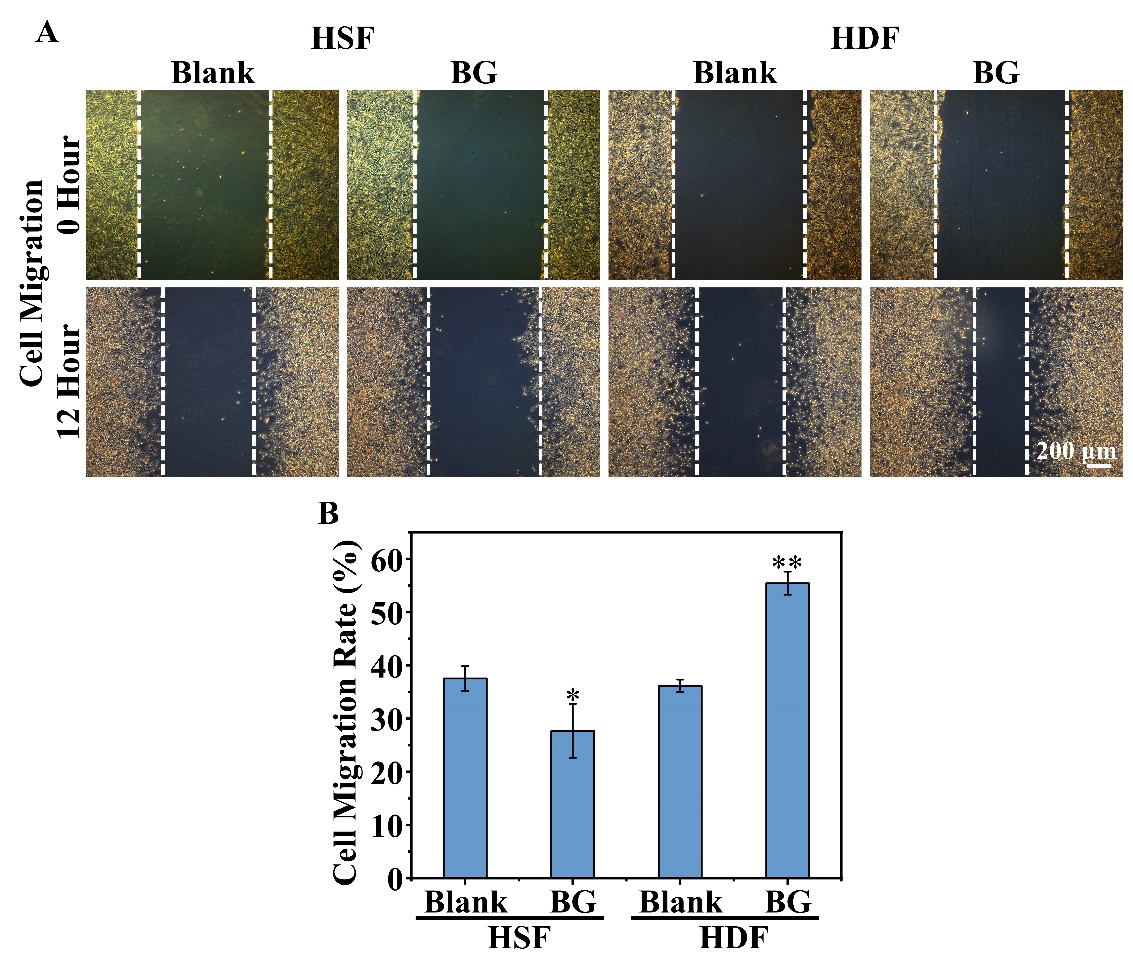


**Figure S5.** Effects of BG on inhibition migration of human scar fibroblasts (HSF) and activation migration of human dermal fibroblasts (HDF). (A) Representative images of cell migration of HSF and HDF with or without treatment with BG. (B) The cell migration rate of HSF and HDF with and without treatment with BG. (***p* < 0.01, ****p* < 0.001 compared with Blank)


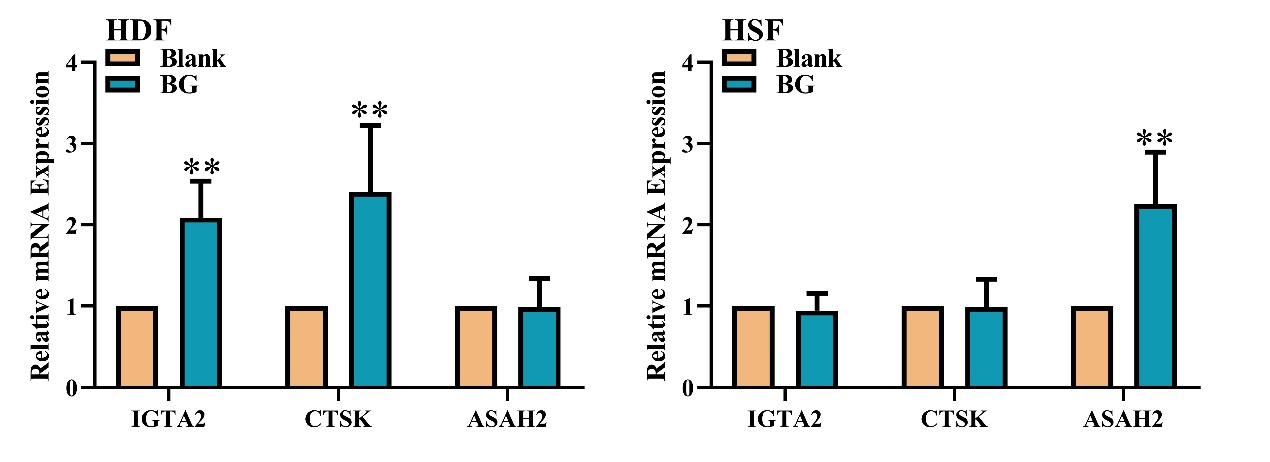


**Figure S6.** qPCR analysis of the expression of representative markers in HDF after the treatment with BG extracts. (**p* < 0.05, ***p* < 0.01 compared with Blank)


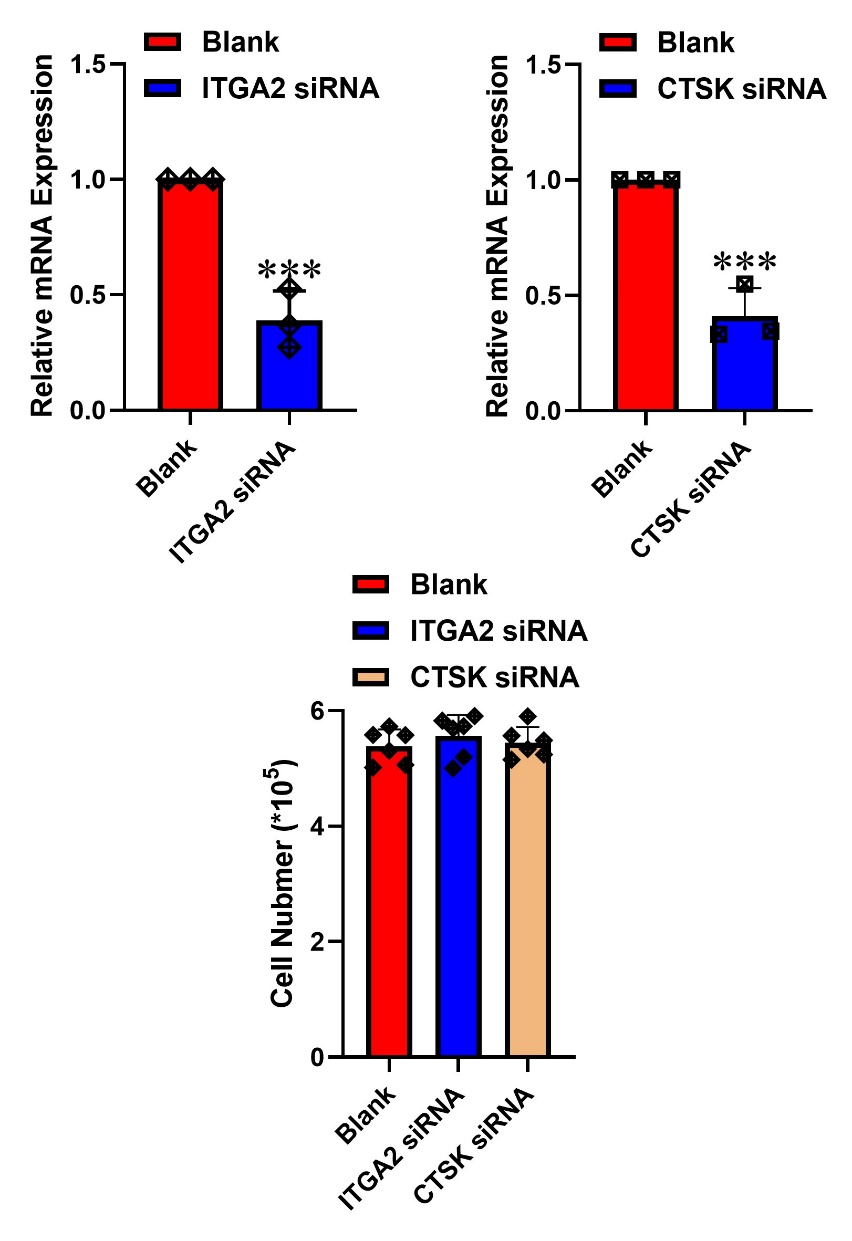


**Figure S7.** qPCR and cell counting analysis demonstrating the knockdown effect and cell viability of ITGA2 siRNA and CTSK siRNA (***p < 0.001 compared with Blank).


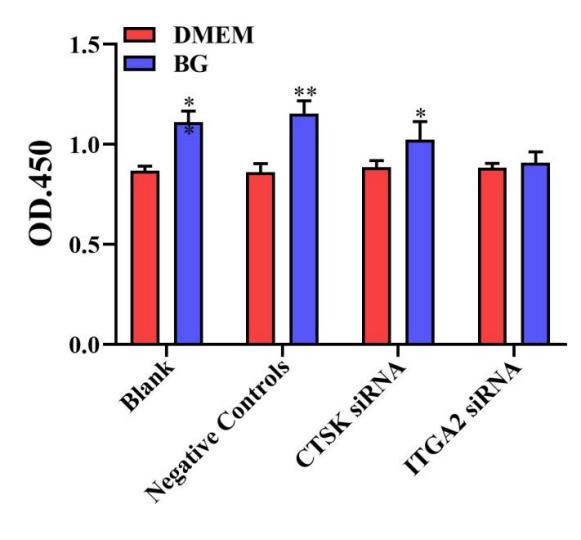


**Figure S8.** Cell viability of HDF treated with BG (HDF were transfected with CTSK siRNA or ITGA2 siRNA.). (**p* < 0.05, ***p* < 0.01 compared with DMEM)


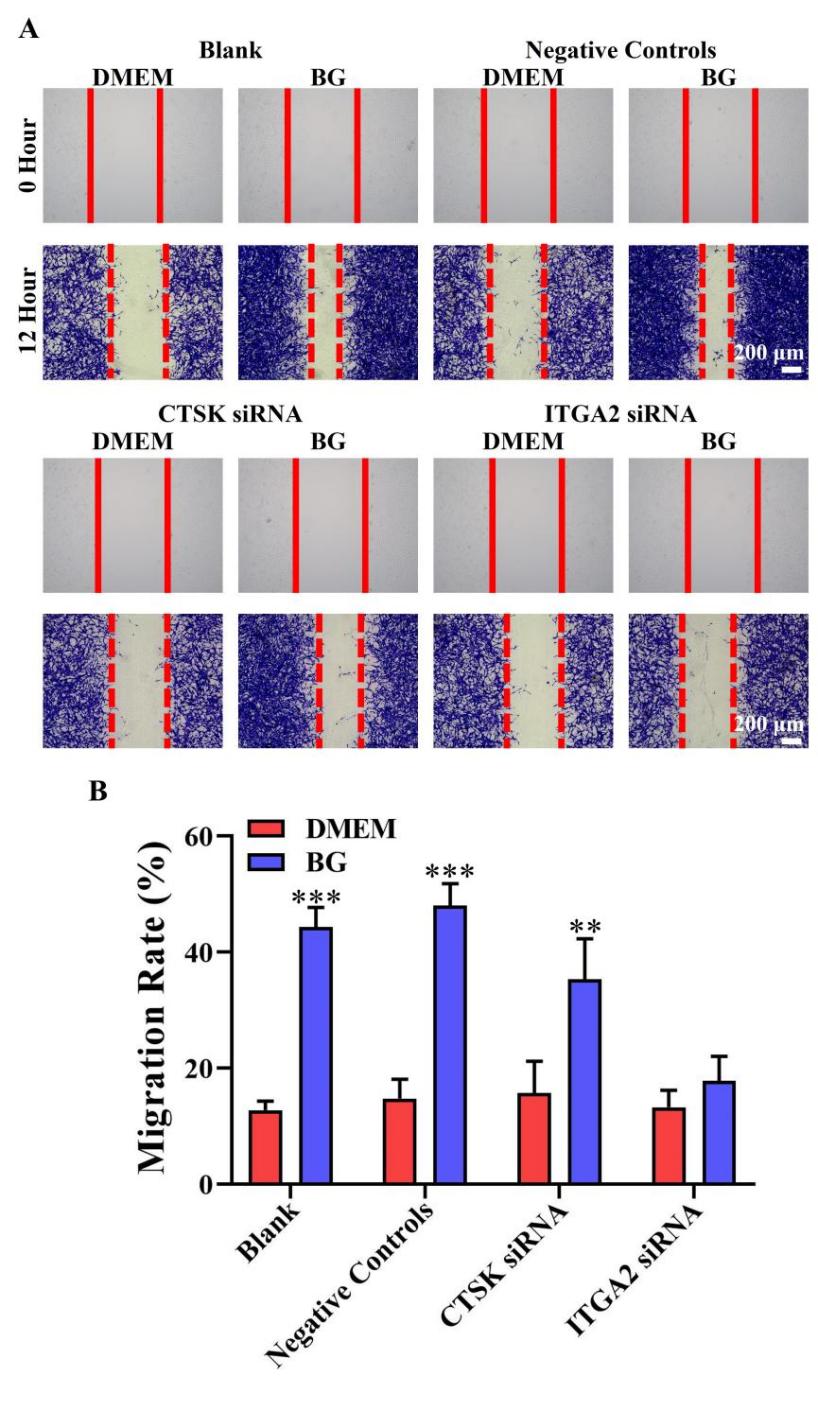


**Figure S9.** (A) Cell migration of HDF treated with BG (HDF were transfected with CTSK siRNA or ITGA2 siRNA.). (B) Quantitative analysis of cell migration rate of HDF. (***p* < 0.01, **p* < 0.001 compared with DMEM)


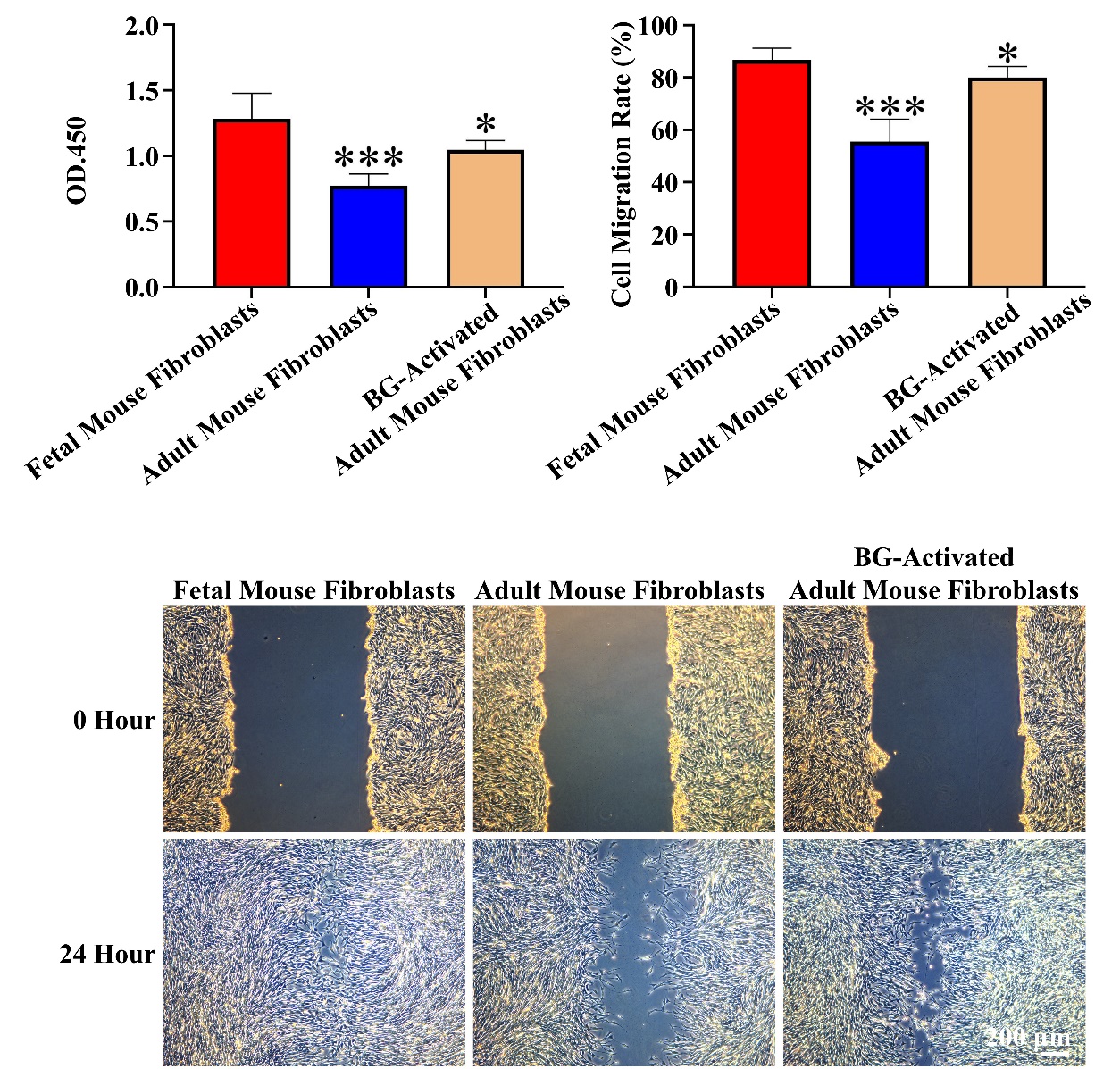


**Figure S10.** Comparison of cell vitality and cell migration between fetal mouse fibroblasts and adult mouse fibroblasts under BG activation. (**p* < 0.05, ****p* < 0.001 compared with Fetal Mouse Fibroblasts)


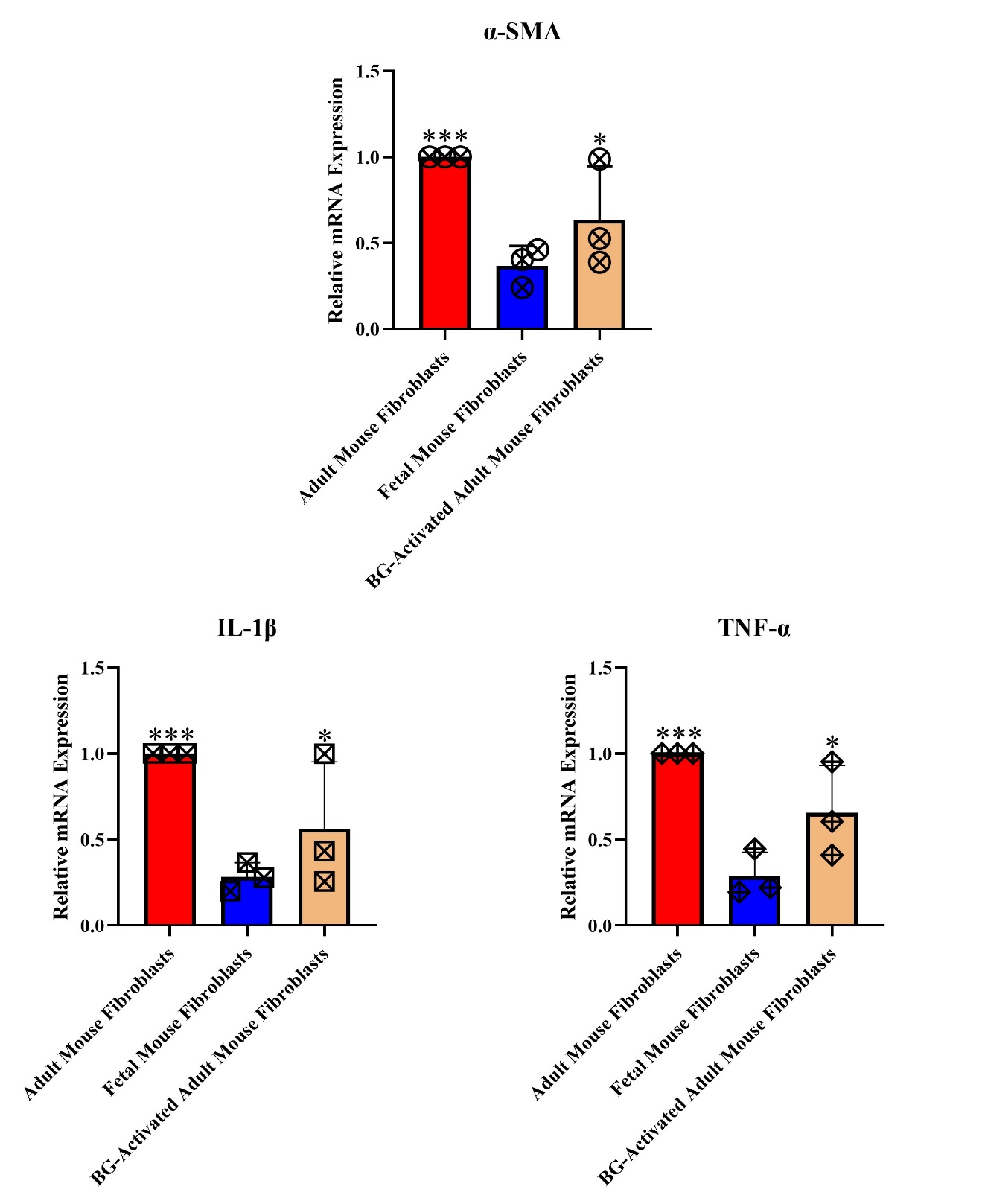


**Figure S11.** Comparison of α-SMA, IL-1β, and TNF-α between fetal mouse fibroblasts and adult mouse fibroblasts under BG activation. (*p < 0.05, ***p < 0.001 compared with Fetal Mouse Fibroblasts)


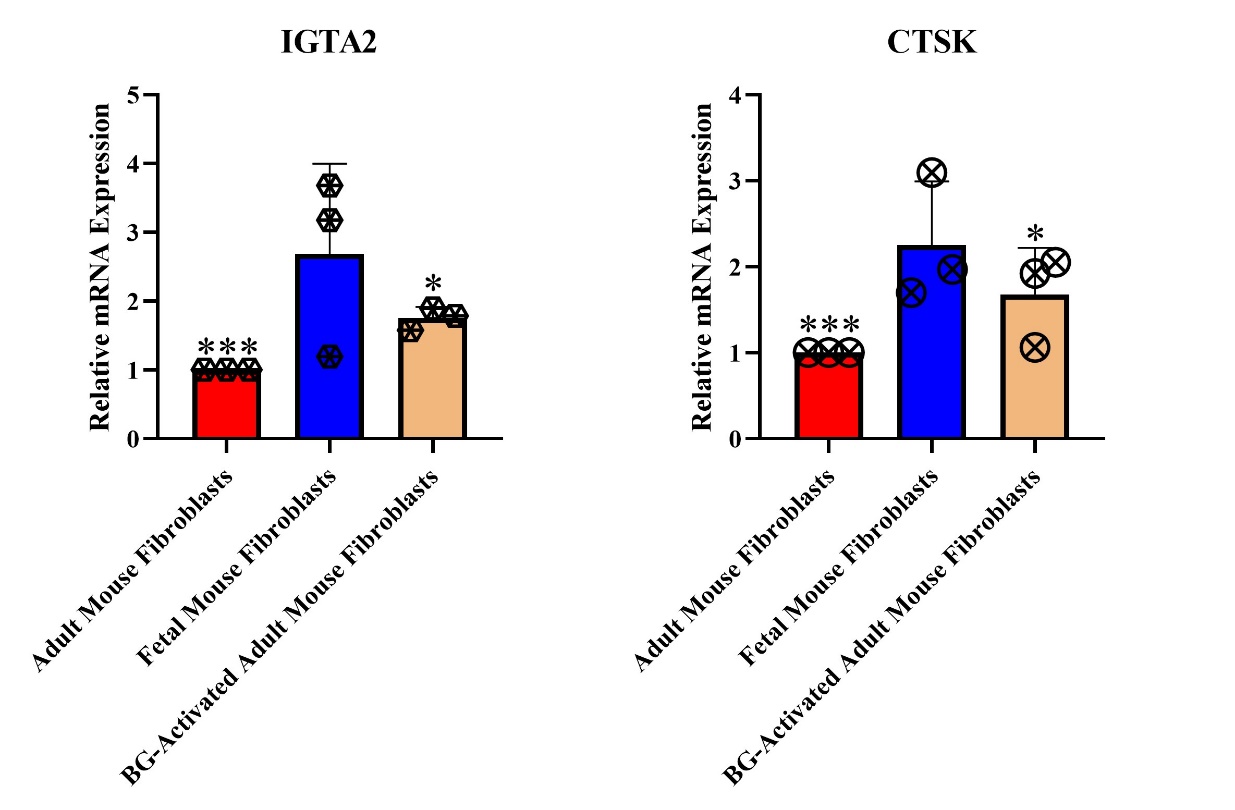


**Figure S12.** Comparison of ITGA2 and CTSK between fetal mouse fibroblasts and adult mouse fibroblasts under BG activation. (*p < 0.05, ***p < 0.001 compared with Fetal Mouse Fibroblasts)


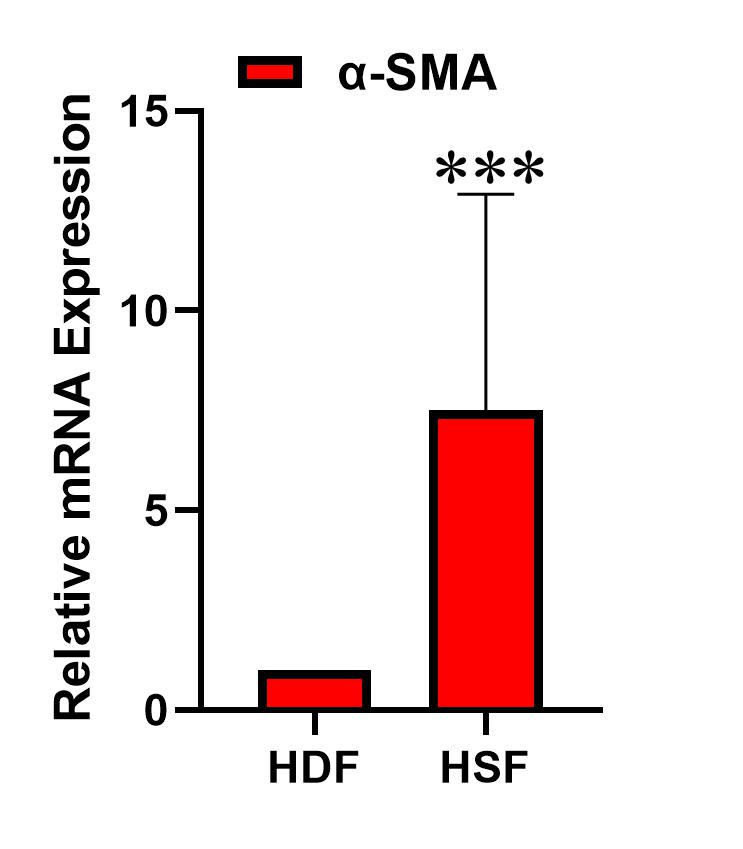


**Figure S13.** qPCR analysis of the HSF phenotype assessed by α-SMA expression in comparison with HDF (***p < 0.001 compared with Blank).


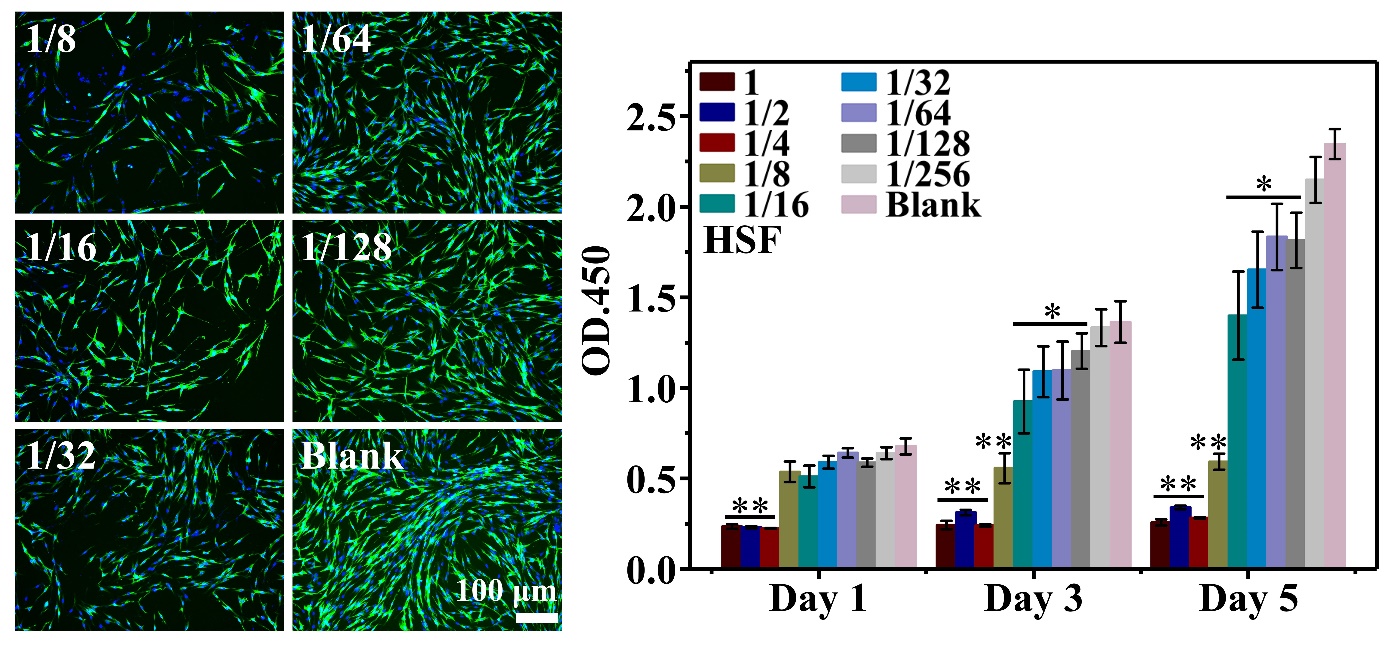


**Figure S14.** BG inhibits HSF viability. The cell viability of HSF cultured with BG extracts (1-1/256) at day 1, 3 and 5. Fluorescent images of HSF treated with BG extract (1/8-1/256) for 48 h. (*p <0.05, **p < 0.01 compared with Blank)


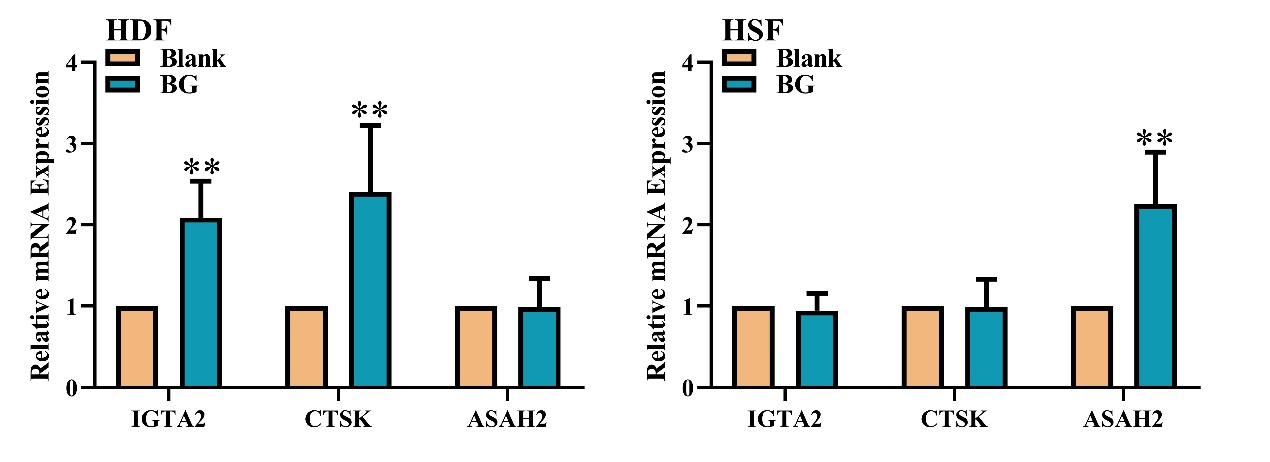


**Figure S15** qPCR analysis of the expression of representative markers in HSF after the treatment with BG extracts. (***p* < 0.01 compared with Blank)


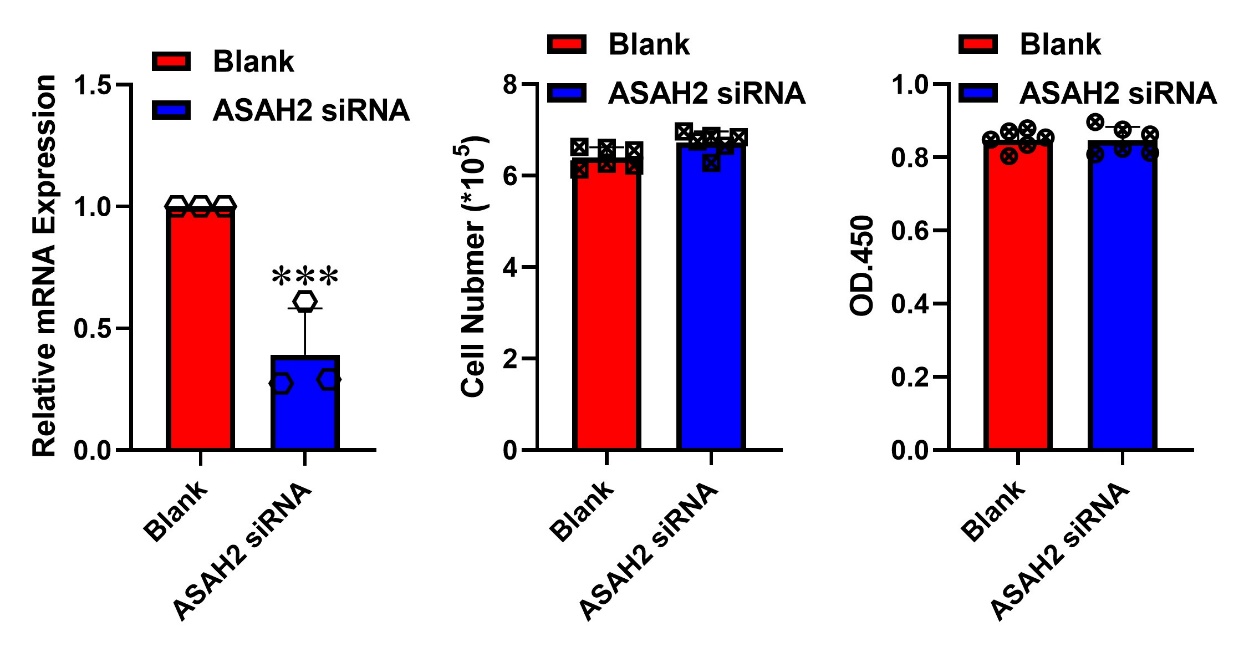


**Figure S16.** qPCR and cell counting and CCK8 analysis demonstrating the knockdown effect and cell viability of ASAH2 siRNA (***p < 0.001 compared with Blank).


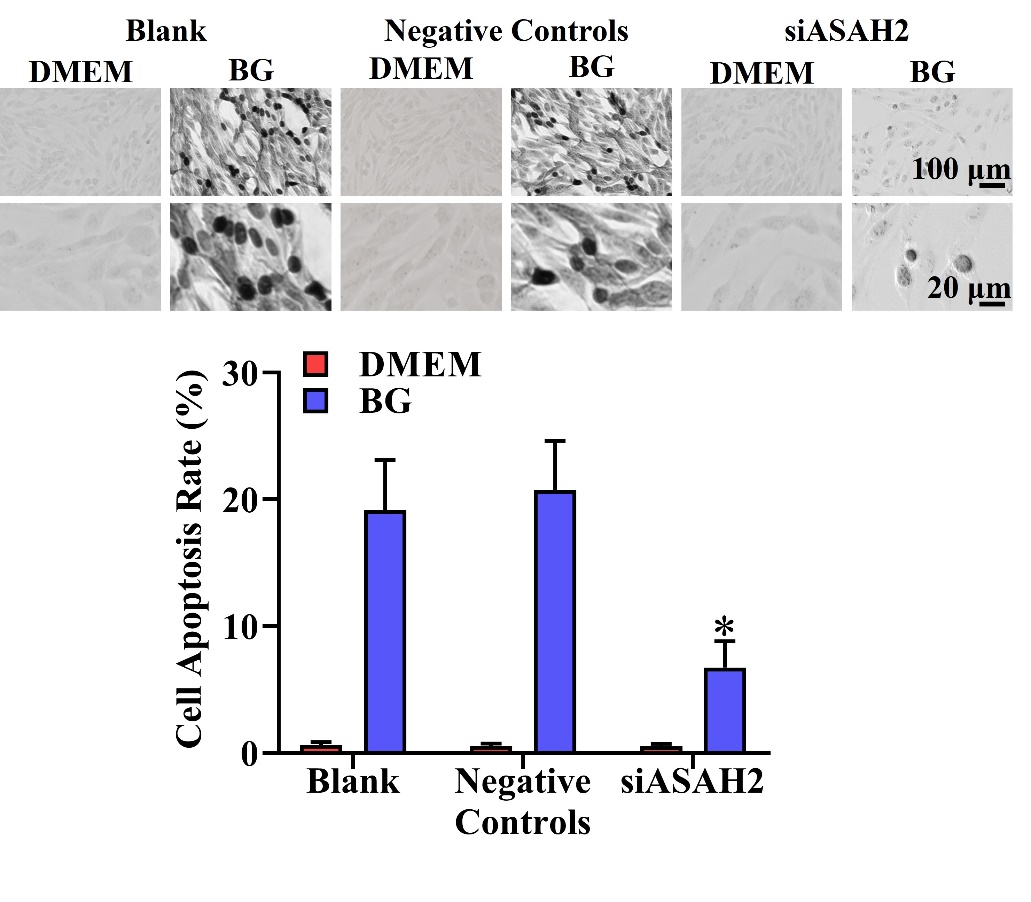


**Figure S17.** Cell apoptosis (TUNEL staining) of HSF treated with BG (HSF were transfected with ASAH2 siRNA.). Quantitative analysis of cell apoptosis rate of HSF treated with BG. (*p < 0.05, compared with DMEM)


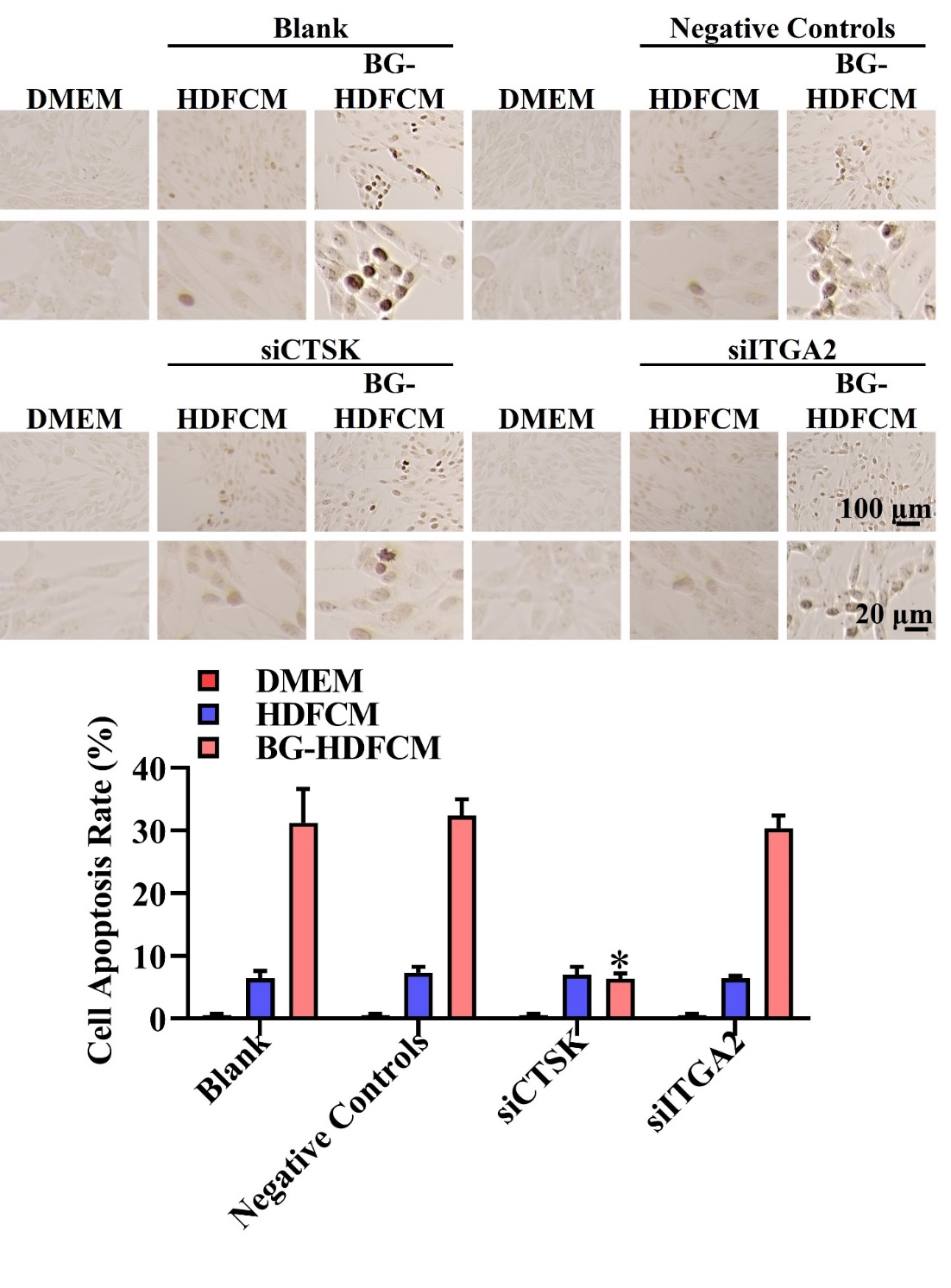


**Figure S18.** Cell apoptosis (TUNEL staining) of HSF treated with conditioned medium acquired from HDF (siCTSK: conditioned medium acquired from HDF transfected with CTSK siRNA; siITGA2: conditioned medium acquired from HDF transfected with ITGA2 siRNA; HDFCM: HDF normal conditioned medium; BG-HDFCM: conditioned medium from HDF cultured with a 1/16 dilution of BG.). Quantitative analysis of cell apoptosis rate of HSF treated with BG-HDFCM. (**p* < 0.05 compared with DMEM)


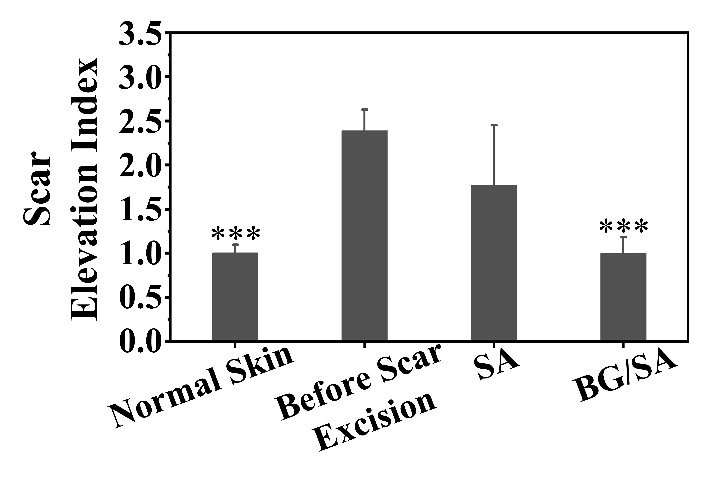


**Figure S19.** Scar elevation index of skin sections of severe rabbit ear scar model. (***p < 0.001, compared with Before Scar Excision)


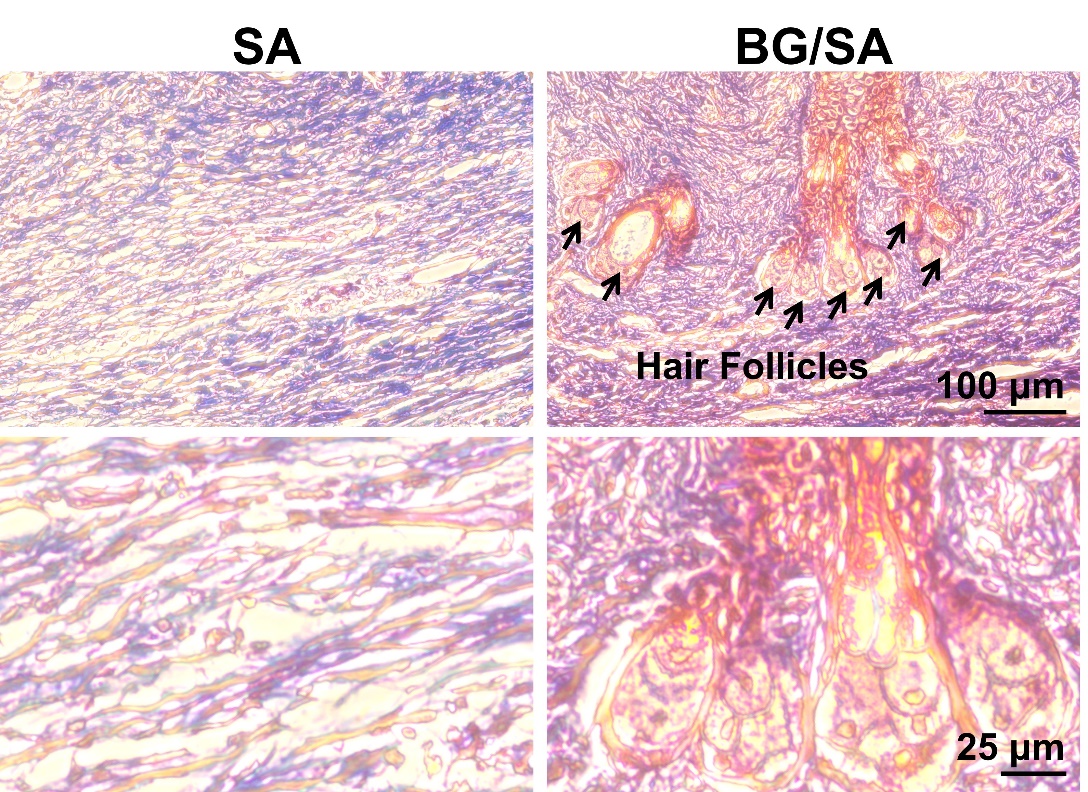


**Figure S20.** Masson staining of skin sections in a severe rabbit ear scar model *in vivo*.

**
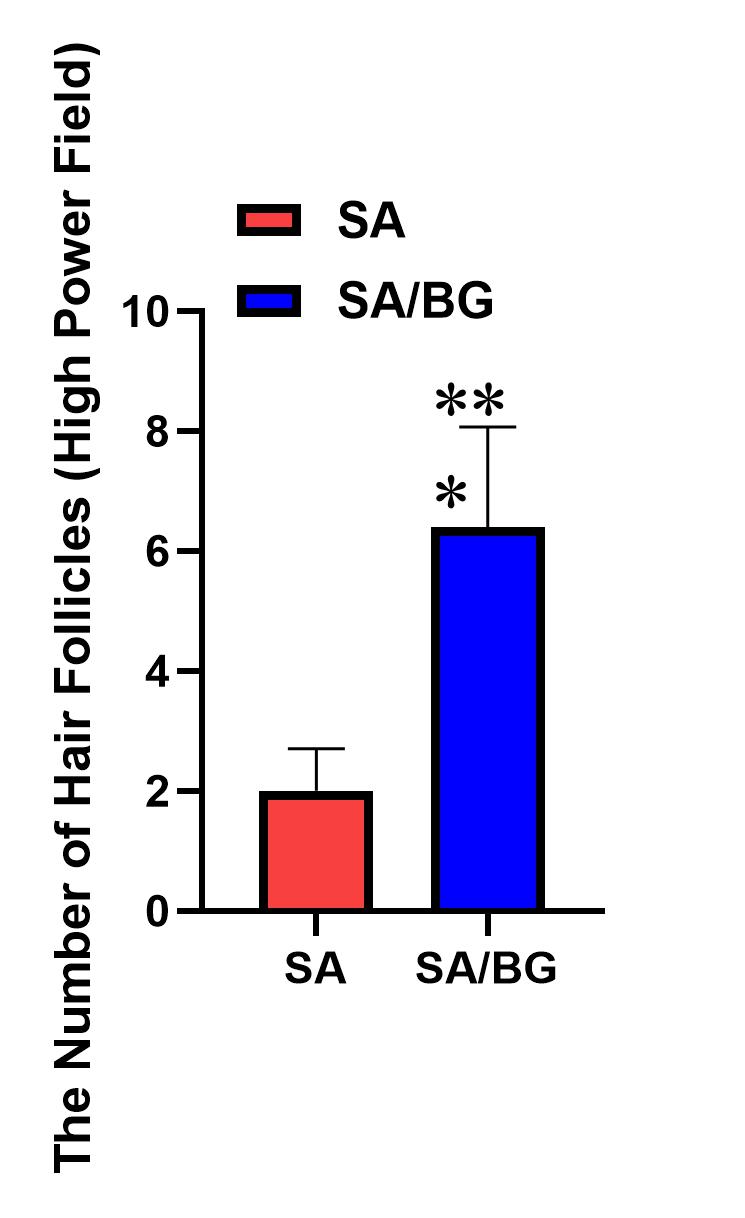
**

**Figure S21.** Quantitative analysis of new born hair follicles in a severe rabbit ear scar model *in vivo* (***p < 0.001 compared with SA).

**
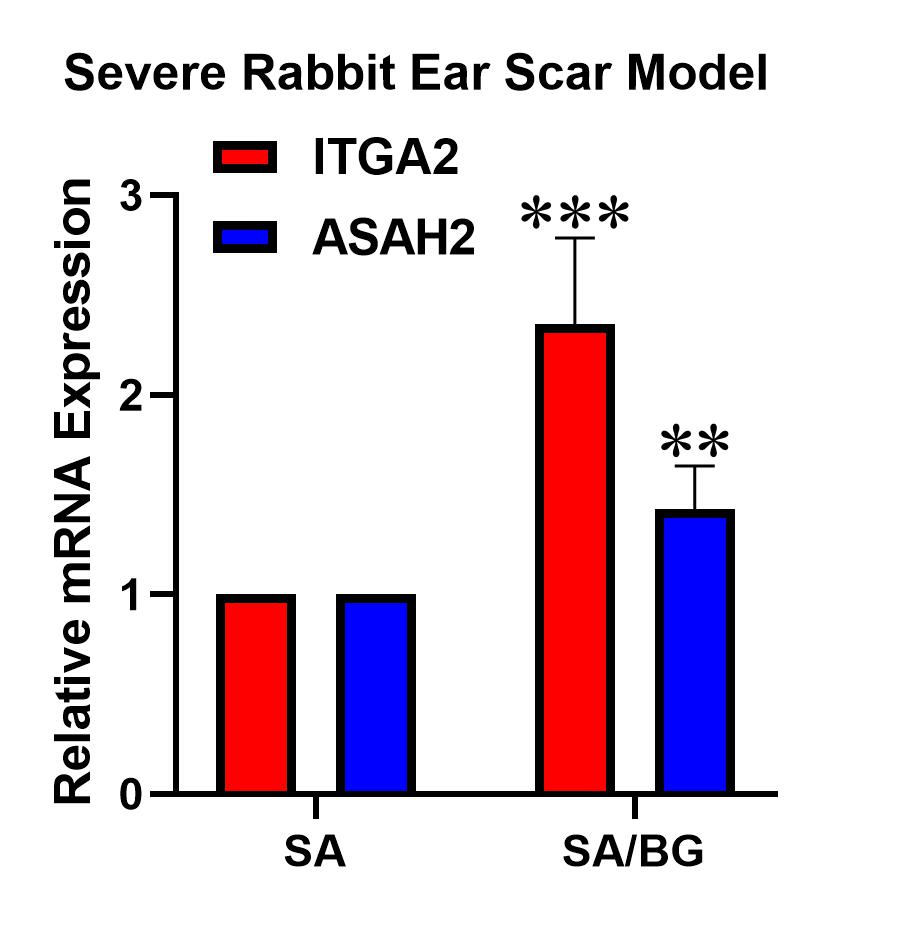
**

**Figure S22.** qPCR analysis of the expression of ITGA2 and ASAH2 on day 56 in the tissue of severe rabbit ear scar model (***p* < 0.01, ****p* < 0.001 compared with SA).

**
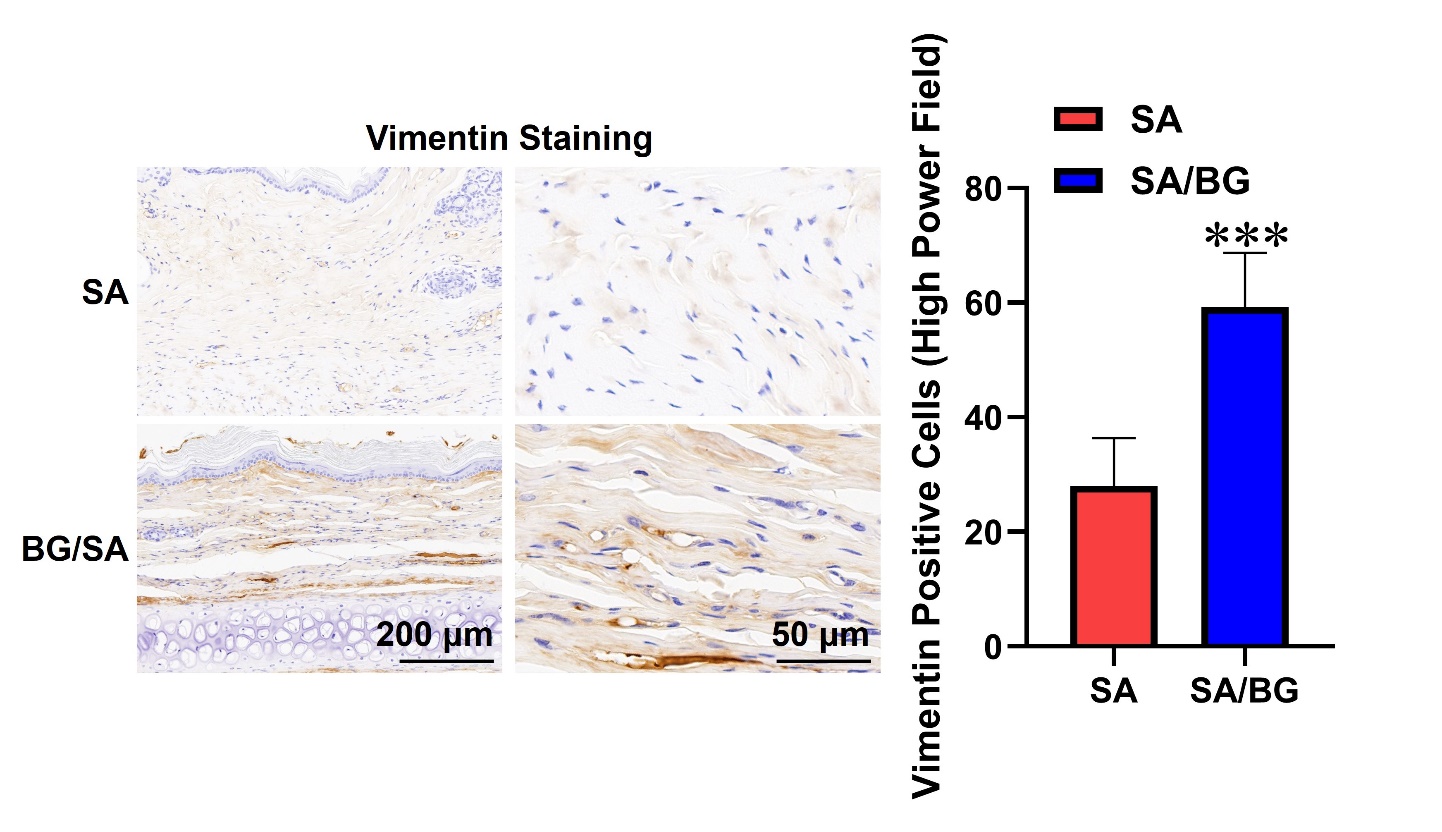
**

**Figure S23.** Vimentin staining of skin sections in a severe rabbit ear scar model *in vivo*. Quantitative analysis of vimentin positive cells (***p < 0.001 compared with SA).


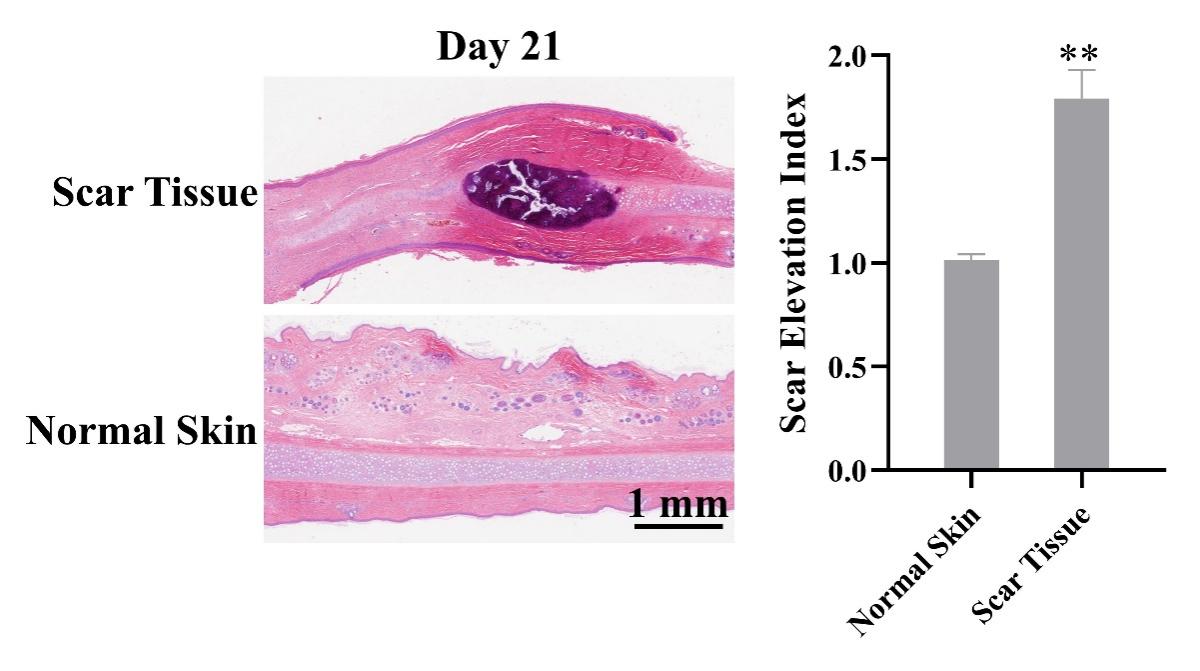


**Figure S24.** H&E staining of scar tissue of acid-corroded rabbit ear hypertrophic scar model and normal skin on day 21.


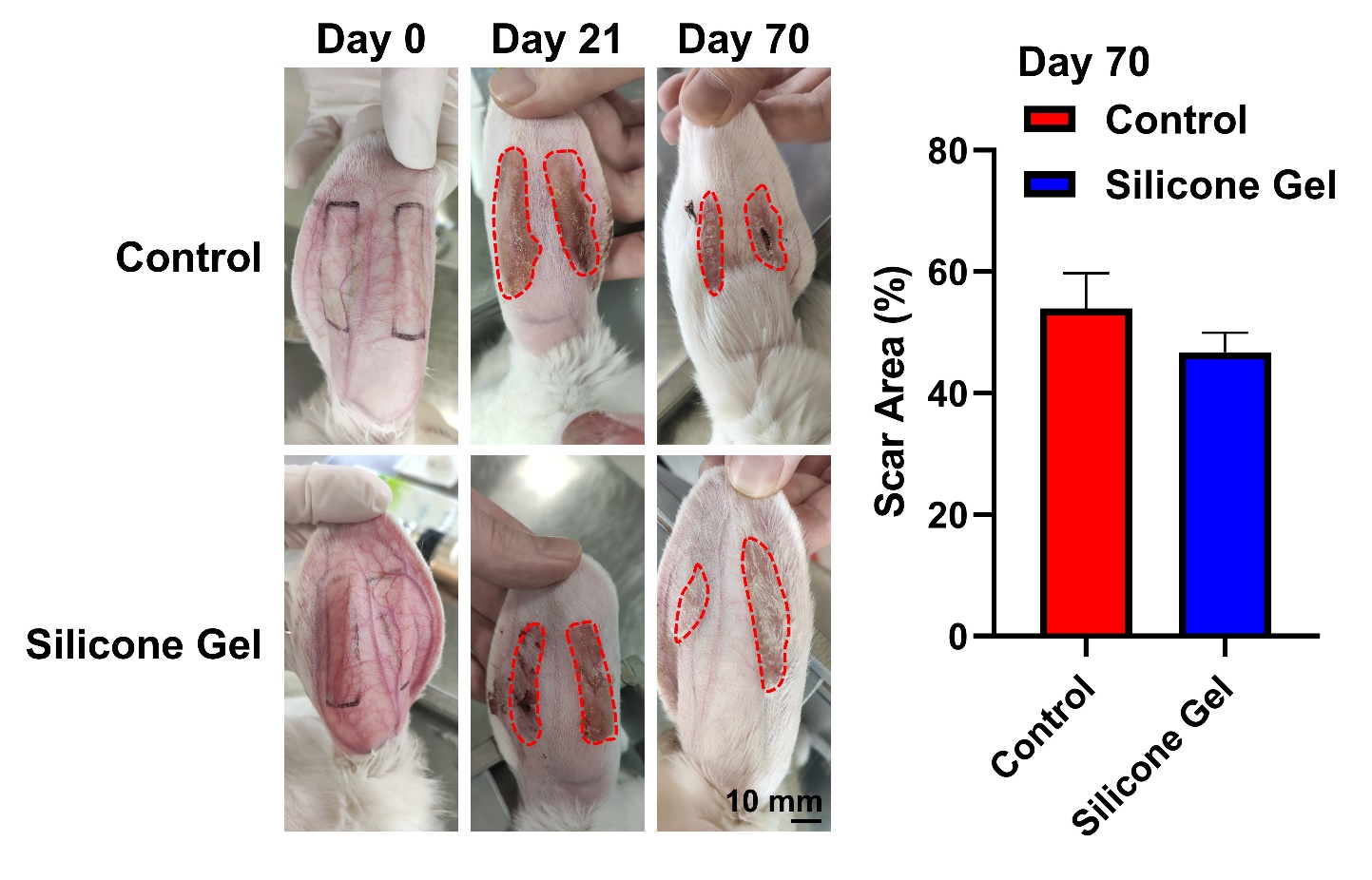


**Figure S25.** Photographs of the scar area following treatment with silicone gel (a commercially available product) and in the untreated injury (Control group) (the red circle indicates the scar area).


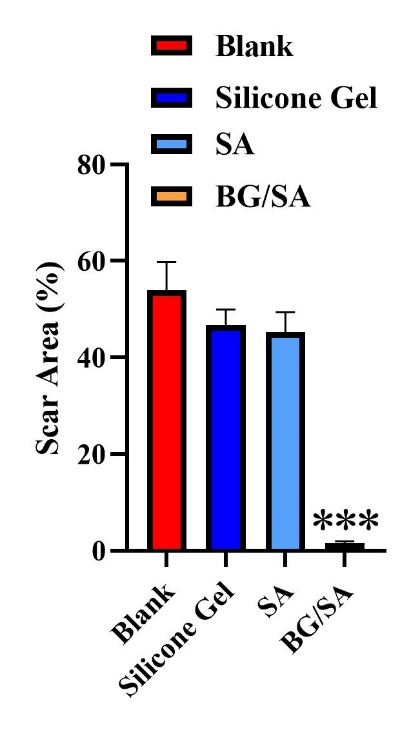


**Figure S26.** The scar area percentage is shown for different treatments. (Control: The wounds injury only; Silicone Gel: The wounds were treated with Silicone Gel from day 22 to day 70; SA: The wounds were treated with SA from day 22 to day 70; BG/SA: The wounds were treated with BG/SA from day 22 to day 70.) (***p < 0.001, compared with Control)


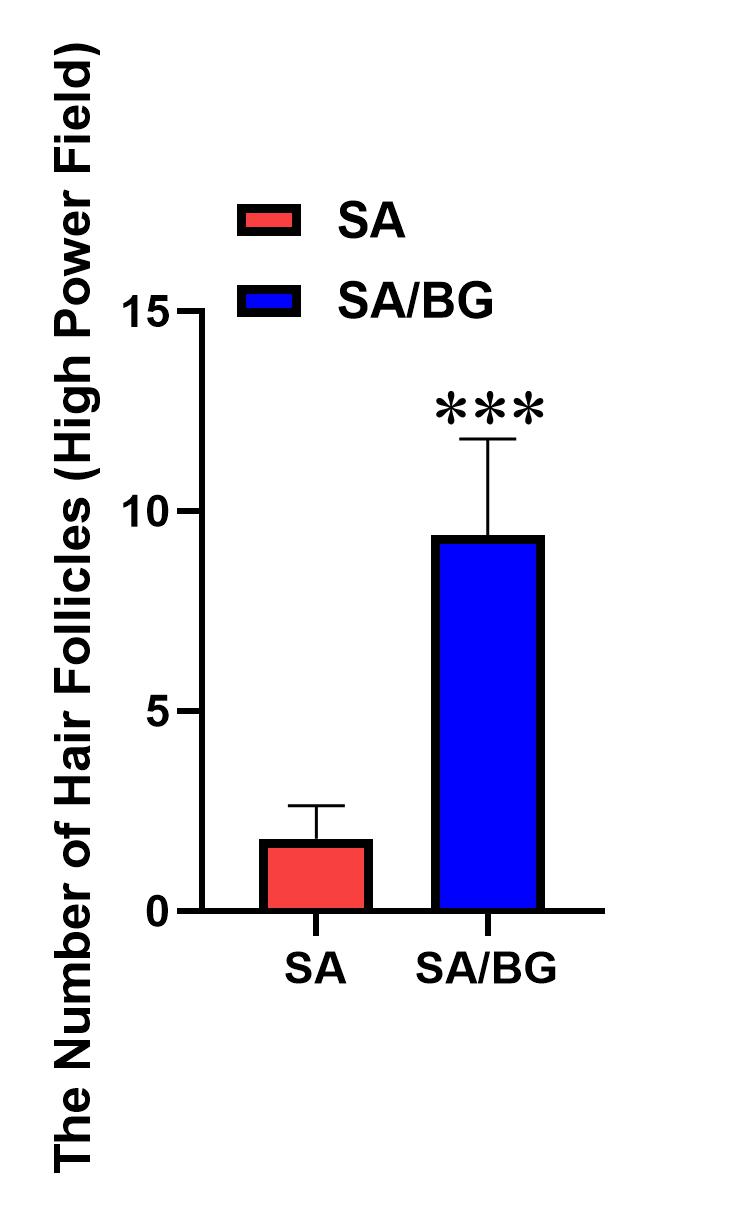


**Figure S27.** Quantitative analysis of new born hair follicles in a mild rabbit ear scar model *in vivo* (***p < 0.001 compared with SA).


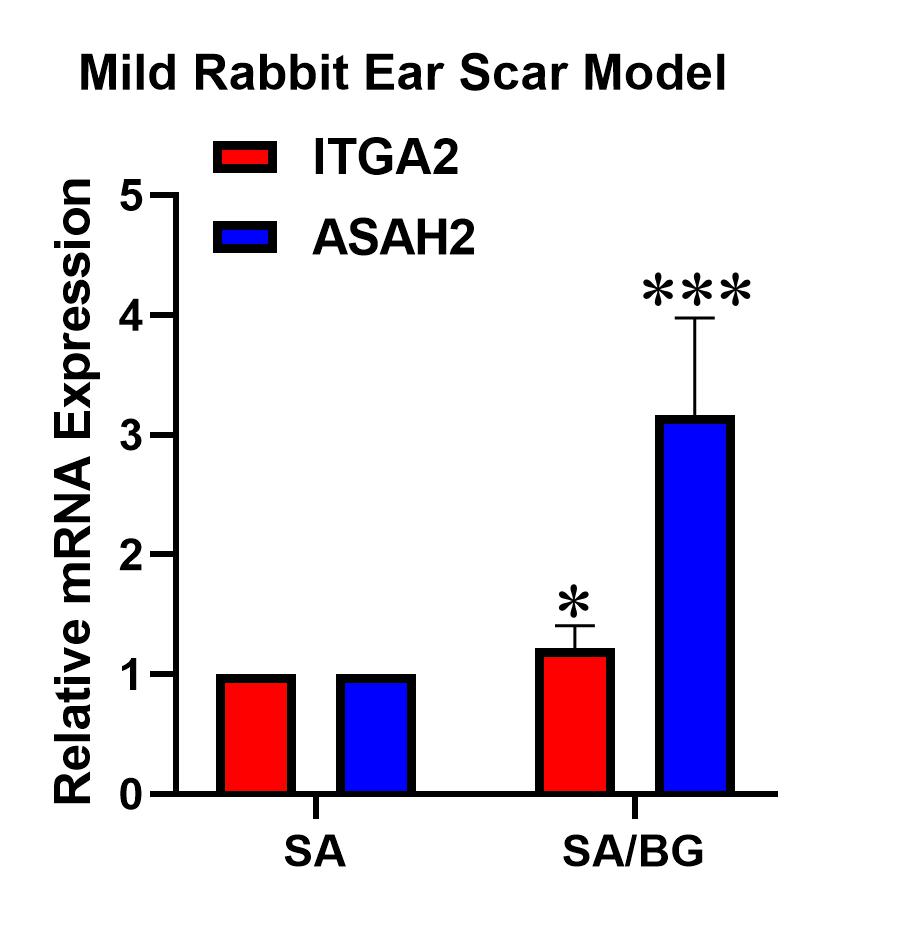


**Figure S28.** qPCR analysis of the expression of ITGA2 and ASAH2 on day 70 in the tissue of mild rabbit ear scar model (**p* < 0.05, ****p* < 0.001 compared with SA).


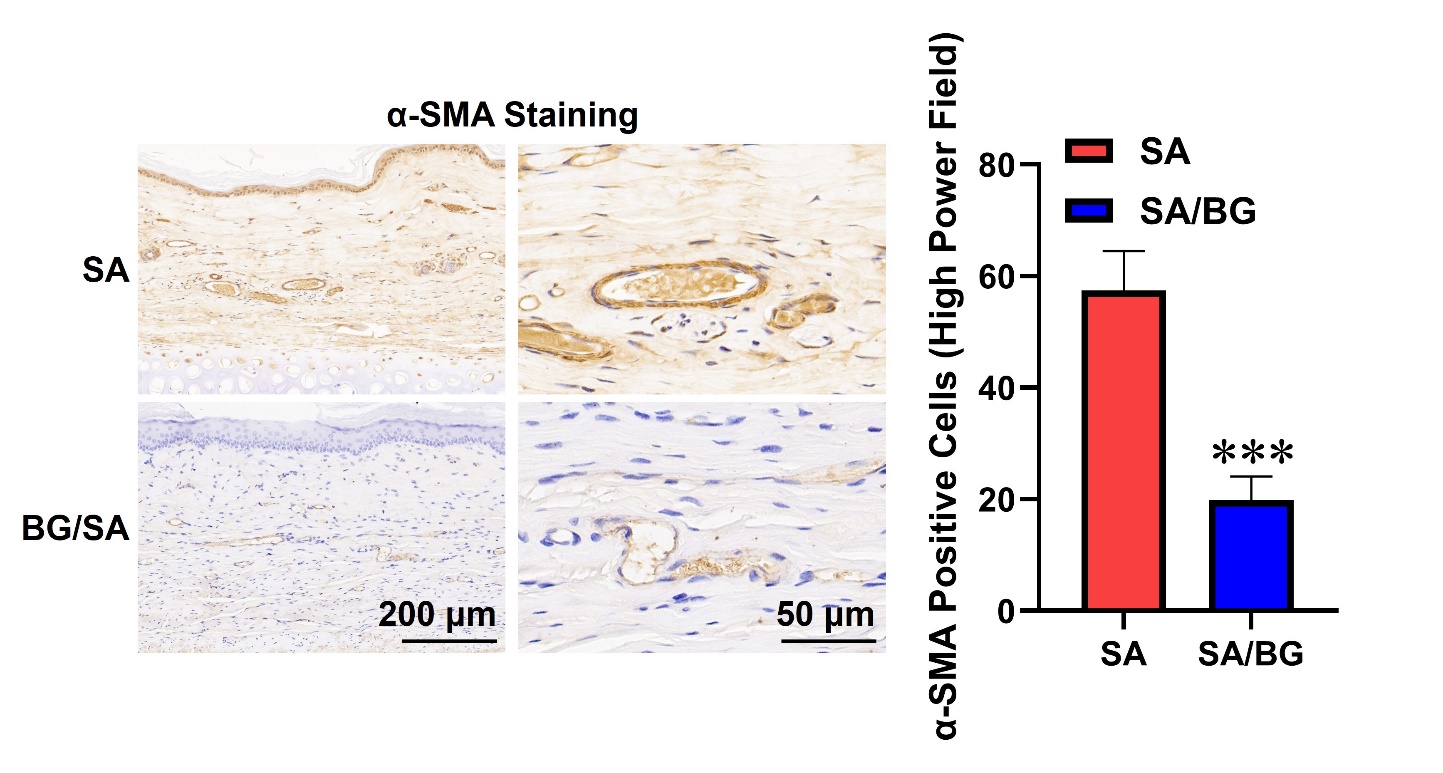


**Figure S29.** α-SMA staining of skin sections in a mild rabbit ear scar model *in vivo*. Quantitative analysis of α-SMA positive cells (***p < 0.001 compared with SA).

**Table S1.** Ion concentrations of different dilution ratios of BG extracts in DMEM medium (1-1/256). 0: DMEM medium without BG extracts.

|  | 1 | 1/2 | 1/4 | 1/8 | 1/16 | 1/32 | 1/64 | 1/128 | 1/256 | 0 |
| --- | --- | --- | --- | --- | --- | --- | --- | --- | --- | --- |
| Ca^2+^ (ppm) | 25.29  ±0.20 | 46.66  ±0.16 | 55.24  ±0.64 | 61.39  ±0.47 | 63.00  ±0.86 | 65.37  ±0.72 | 65.98  ±0.43 | 67.98  ±0.88 | 68.71  ±0.19 | 68.51  ±1.18 |
| SiO_3_^2-^  (ppm) | 820.44  ±4.89 | 411.54  ±2.37 | 202.41  ±0.84 | 102.78  ±0.19 | 51.13  ±0.92 | 25.97  ±0.07 | 13.03  ±0.14 | 6.69  ±0.11 | 3.50  ±0.08 | 0.14  ±0.02 |

**Table S2.** Primer sequences for the genes used in this study.

| Gene | Forward primer | | Reverse primer |
| --- | --- | --- | --- |
| ITGA2 (Human) | 5’- GTGGTCCTCTGTGGGCACAGC-3’ | 5’-TGAGGGAAGGGCAGGGCTGA-3’ | |
| CTSK  (Human) | 5’-ACTTGGGAGACATGACCAGTGA-3’ | 5’-TCTTGACTGGAGTAACGTATCCTTTC-3’ | |
| ASAH2  (Human) | 5’-GGATGGCGATGGTCAGGAAA-3’ | 5’-TTGGAGAGAGTTGCTTGGTG-3’ | |
| α-SMA (Human) | 5’-CCGACCGAATGCAGAAGGA-3’ | 5’-ACAGAGTATTTGCGCTCCGAA-3’ | |
| β-actin  (Human) | 5’-AGAGGGAAATCGTGCGTGAC-3’ | 5’-CAATAGTGATGACCTGGCCGT-3’ | |
| ITGA2 (Rabbit) | 5'-CCTTGGATGGCTATGGAGATTTA-3' | 5'-GACCTTGGCAGTCTCAGAATAG-3' | |
| ASAH2  (Rabbit) | 5’-CACAGATCAACCGAAGCCCTTA-3’ | 5’-CGTCTCCATTCAAATCCACCAT-3’ | |
| GAPDH  (Rabbit) | 5’-TCACCATCTTCCAGGAGCGA-3’ | 5’-CACAATGCCGAAGTGGTCGT-3’ | |
| α-SMA (Mouse) | 5’-CGGCTTCGCTGGTGATGA-3’ | 5’-TCCCTCTCTTGCTCTGGGCTT-3’ | |
| IL-1β  (Mouse) | 5’-CTACCTGTGTCTTTCCCGTG-3’ | 5’-TTTGTTGTTCATCTCGGAGC-3’ | |
| TNF-α  (Mouse) | 5’-CTGTAGCCCACGTCGTAGCAA-3’ | 5’-TGTCTTTGAGATCCATGCCGTT-3’ | |
| ITGA2 (Mouse) | 5’-GTTGCTTGCACGTTGATATGG-3’ | 5’-CAATATCCAGGTACACATCAACAC-3’ | |
| CTSK  (Mouse) | 5’-CCAGGAAATGAGCTTGACAAA-3’ | 5’-ATAATTCTCAGTCACACAGTCCACA-3’ | |
| GAPDH  (Mouse) | 5’-AGAACATCATCCCTGCATCCAC-3’ | 5’-TCAGATCCACGACGGACACA-3’ | |
